# Can Bureaucrats Really Be Paid Like Ceos? Substitution Between Incentives and Resources Among School Administrators in China

**DOI:** 10.1093/jeea/jvy047

**Published:** 2019-01-17

**Authors:** Renfu Luo, Grant Miller, Scott Rozelle, Sean Sylvia, Marcos Vera-Hernández

**Affiliations:** 1 Peking University; 2 Stanford University; 3 University of North Carolina, Chapel Hill; 4 University College London

## Abstract

Unlike performance incentives for private sector managers, little is known about performance incentives for managers in public sector bureaucracies. Through a randomized trial in rural China, we study performance incentives rewarding school administrators for reducing student anemia—as well as complementarity between incentives and orthogonally assigned discretionary resources. Large (but not small) incentives and unrestricted grants both reduced anemia, but incentives were more cost-effective. Although unrestricted grants and small incentives do not interact, grants fully crowd-out the effect of larger incentives. Our findings suggest that performance incentives can be effective in bureaucratic environments, but they are not complementary to discretionary resources.

## 1. Introduction

The provision of public services in many developing countries is low in quality (Banerjee, Deaton, and Duflo 2004; World Bank 2004; Das, Hammer, and Leonard 2008; Berendes et al. 2011). Although the underlying reasons are complex and incompletely understood, the culprit is not simply lack of resources, inadequate training, or deficiencies in provider knowledge. Supply-side incentives are also often poorly aligned with social objectives. Absenteeism in many parts of the world is pervasive (Chaudhury and Hammer 2004; Kremer et al. 2005; Banerjee and Duflo 2006; Chaudhury et al. 2006; Lewis 2006), and providers often fail to do in practice what is within their knowledge and means (Das and Hammer 2004; Alcázar et al. 2006; Chaudhury et al. 2006; Das and Hammer 2007; Leonard and Masatu 2010; Das et al. 2012; Sylvia et al. 2015).

To better align provider incentives with social objectives, performance pay has become increasingly common in public sector service delivery (Oxman and Fretheim 2008; Eichler and Levine 2009; Miller and Babiarz 2014). Drawing on the logic of performance pay in human resource management (Lazear 1995; Hall and Liebman 1998; Lazear 2000), this approach provides direct financial rewards for achieving prespecified performance targets. Despite its growing prominence, however, there is remarkably little empirical evidence on basic mechanism design considerations in the use of performance pay to improve public service delivery (Miller and Babiarz 2014).

This paper contributes to the literature on performance pay in developing countries—and in particular, its design—through a large-scale experiment studying the interaction of performance incentives and unconditional block grants, both of varying sizes, for public sector administrators. Specifically, we provide primary school administrators (lead principals who are the managers and executive decision-makers in schools—hereafter “administrators”) with randomly assigned budget transfers (large and small) and randomly assigned financial incentives (large and small) for improving the health of their students. Our health focus is anemia, a leading child health problem in rural China.^1^

Our study yields four key findings. First, we find that larger incentives for anemia reduction were effective when administrators had fewer resources at their disposal for implementing the program. Incentives that provided substantial additional income to administrators (mean realized payouts of about 2 months of annual salary) reduced anemia among students who were anemic at baseline by 13.8 percentage points (or 38%). Second, in contrast, small incentives (one tenth the size of the larger incentives) were ineffective in reducing anemia—and were significantly less effective than large incentives. Third, even absent explicit incentives, unrestricted budget transfers to school administrators led to sizeable reductions in anemia, suggesting other motives among administrators to allocate resources toward student nutrition. However, the resource cost of reducing anemia through larger school budgets was approximately twice as great (per case of anemia averted) as combining larger performance incentives with smaller budgets—implying that school administrators with explicit financial incentives used smaller budgets with greater productive efficiency.

Finally, we find that explicit financial incentives and unrestricted grants can be strong substitutes—and whether or not they are depends critically on incentive size. Specifically, we find that large incentives and grants are pronounced substitutes. The effect is particularly strong: unrestricted resource transfers (of sizes chosen by government planners in practice) crowd-out the effect of large incentives. Importantly, we find this pattern of results not only with student nutritional outcomes, but also with intermediate measures of resource allocation and effort (e.g., the provision of better nutrition and effort to persuade parents to improve their children's diets at home). Substitution therefore reflects reductions in administrator effort with larger budgets (and not simply decreasing marginal returns to inputs in the biological production of child nutrition).^2^

Our findings contribute to existing literature on performance incentives in several ways. First, previous studies on the role of performance pay in the public sector have generally focused on front-line workers rather than public sector managers (or “bureaucrats”—an exception is Rasul and Rogger 2018).^3^ However, the scope of behavioral responses among managers is potentially much broader, possibly with greater potential for improving public sector service delivery. Specifically, rather than simply increasing effort, the actions of managers can have greater influence on productive and allocative efficiency because of the resources under over which they have decision-making authority (Holmstrom and Ricart i Costa 1986; Athey and Roberts 2001; Bandiera, Barankay, and Rasul 2007; Burgess et al. 2010).

Second, existing studies of performance pay for managers generally examine the private sector, but insights from this literature cannot be easily extrapolated to bureaucracies.^4^ Career concerns can be particularly strong in bureaucracies—and they may overpower or interact with incentives created by performance pay (Gibbons and Murphy 1992). Moreover, civil servants may be considerably more prosocially or intrinsically motivated (Francois 2000; Francois and Vlassopoulos 2008; Tonin and Vlassopoulos 2015)—and performance pay may dampen the effects of these motivations (see Fehr and Falk 2002; Gneezy, Meier, and Rey-Biel 2011; Kamenica 2012 for reviews). Finally, public sector production processes tend to be both more complex (due to multiple objectives and multiple agents—Dixit (2002)) and more heterogeneous (due to a primary goal being to expand access, which necessitates operation in a wider range of contexts than private sector organizations that have more scope to select the markets in which they operate). Performance pay may therefore be ineffective if rewards are not well-aligned with effective inputs across the range of contexts within which an organization operates.

Third, we contribute to existing literature by studying three mechanism design considerations of performance pay. One is that we reward outputs directly. In contrast to rewarding inputs, performance incentives for outputs strengthens incentives for managers to draw on local information and contextual knowledge to improve both allocative and productive efficiency—or to “innovate.” Our study is one of the first focused on health to reward health outputs—and we do indeed find evidence of managerial innovation (school administrators successfully work with parents to improve diets at home).^5^ Another is that we directly study differential behavioral responses to performance incentives of varying sizes. Existing literature on this issue is split: a number of studies outside of organizational settings report large responses to very modest rewards (as well as highly elastic demand at prices close to zero),^6^ whereas others suggest small responses—or even reductions in effort (e.g. when intrinsic motivation is crowded-out).^7^ Our results are more closely aligned with the latter.^8^ Finally, we provide first evidence on how incentives interact with the amount of resources under contracted agents’ control. A common focus is on the relative effects of incentive and resource-based approaches (see Lavy 2002; Hanushek 2006); however, these two approaches are often implemented simultaneously and are likely to interact in important ways. Theoretically, incentives and resources available to managers can be complements or substitutes. We study this issue both theoretically and empirically, find evidence of strong substitution when incentives and budgets are large.

The rest of this paper is organized as follows. Section 2 presents a conceptual framework for understanding school administrators’ behavioral responses to output-based performance incentives. Section 3 provides background on school-based nutrition programs as well as the causes and consequences of anemia. Section 4 describes our experimental design, data collection, and methods. Section 5 reports our results, and Section 6 concludes.

## 2. Conceptual Framework

In this section, we propose a simple model of the school administrator decision problem that we study. Specifically, we consider the influence of both output-based performance incentives and discretionary resources on organizational effort—as well as how they interact (i.e., if they are substitutes or complements). We model the school administrator (bureaucrat) as choosing effort *e* to reduce anemia in the school.^9^ Additionally, the school administrator also decides on the allocation of resources—in particular, how to divide the school budget *G* between anemia reduction *A* and other school functions *G* − *A*. The health production function *f*(*e*,  *A*) combines the school administrator's effort *e* and the funds allocated to reducing anemia *A* in determining student health *H*. The school administrator's maximization problem is therefore

(1) \begin{equation*} \mathop {\max }\limits_{e,A} \ \quad w + \theta H - v\left( e \right) + S\left( {G - A} \right) \end{equation*}

(2) \begin{equation*} {\rm subj.\ to{:}}\ \quad w = tH + m, \end{equation*}

(3) \begin{equation*} H = f\left( {e,A} \right), \end{equation*}

(4) \begin{equation*} G \le A \end{equation*}

Total take-home pay $w$ includes both base pay *m* and a reward or bonus for improving student health, *tH*, which is the product of *t*, the marginal bonus, and *H*, the net gain in student health (in our case, the net reduction in the number of students with anemia). Disutility of effort, $v$(*e*), is also strictly increasing but convex: $v$΄ > 0,  $v$΄ ≥ 0. The parameter θ, which is non-negative, allows the school administrator to be altruistic, deriving direct utility from student health (prosociality and public service motivation are often considered important among public sector workers—e.g., see Besley and Ghatak 2007; Dal Bó, Finan, and Rossi 2013). The school administrator also derives utility from school functions unrelated to health, *S*(*G* − *A)*, which is also assumed to be increasing (*S*΄ > 0) and concave (*S*΄ ≤ 0). We make standard assumptions that the health production function, *f*(*e*,  *A*), is increasing in both arguments and concave (*f_e_* > 0,  *f_ee_* < 0, *f_A_* > 0, *f_AA_* < 0,  *f_ee_  f_AA_* − *f_eA_* ≥ 0) and the intuitive assumption that *f_eA_* ≥ 0, or that the marginal productivity of one input is nondecreasing in the level of the other input.

Assuming an interior solution, the solution to (1)–(4) is equivalent to the solution to

(5) \begin{equation*} \mathop {\max }\limits_{e,A} \ \left( {\theta + t} \right)f\left( {e,A} \right) - v\left( e \right) + S\left( {G - A} \right). \end{equation*}

The first order conditions are

(6) \begin{equation*} {U_e} \equiv \left( {\theta + t} \right){f_e}\left( {e,A} \right) - v'\left( e \right) = 0,\phantom{-AA} \end{equation*}

(7) \begin{equation*} {U_A} \equiv \left( {\theta + t} \right){f_A}\left( {e,A} \right) - S'\left( {G - A} \right) = 0. \end{equation*}

The first order condition (6) implies that the optimal level of effort equates the marginal benefit of increasing effort (the increase in health, *f_e_*(*e*, *A*), multiplied by *t* + θ, reflecting both the increase in take-home pay and the altruistic increase in direct utility) with its marginal cost. Equivalently, the first order condition (7) implies that resources *G* are invested in activities unrelated to nutrition up to the point that its marginal benefit, *S*΄(*G* − *A*), equals the marginal benefit of investing in nutrition-related activities, (θ + *t*)*f_A_*(*e*, *A*).

The second order conditions required for a maximum are

(8) \begin{equation*} {U_{ee}} \equiv \left( {\theta + t} \right){f_{ee}}\left( {e,A} \right) - v'' < 0, \end{equation*}

(9) \begin{equation*} {U_{AA}} \equiv \left( {\theta + t} \right){f_{AA}}\left( {e,A} \right) + S'' < 0, \end{equation*}

(10) \begin{equation*} \left| H \right| \equiv {U_{ee}}{U_{AA}} - U_{eA}^2 > 0,\quad {\rm{where}}\quad {U_{eA}} \equiv \left( {\theta + t} \right){f_{eA}}. \end{equation*}

### 2.1. Comparative Statics

We analyze how the school administrator's choice of effort and resources dedicated to nutrition changes both with incentives *t* and discretionary resources *G*—both separately and in combination (as we study empirically through our experiment). First, we consider each effect separately; the corresponding first order comparative statics (see the Online Appendix B for these and other derivations and proofs) are

(11) \begin{equation*} \frac{{de}}{{dt}} = \frac{{ - {f_e}\left[ {\left( {\theta + t} \right){f_{AA}} + S''} \right] + \left( {\theta + t} \right){f_A}{f_{eA}}}}{{\left| H \right|}} > 0, \end{equation*}

(12) \begin{equation*} \frac{{dA}}{{dt}} = \frac{{ - {f_A}\left[ {\left( {\theta + t} \right){f_{ee}} + v''} \right] + \left( {\theta + t} \right){f_e}{f_{eA}}}}{{\left| H \right|}} > 0,{\rm{\ }} \end{equation*}

(13) \begin{equation*} \frac{{de}}{{dG}} = \frac{{ - \left( {\theta + t} \right){f_{eA}}S''}}{{\left| H \right|}} > 0,\phantom{S1A} \end{equation*}

(14) \begin{equation*} \frac{{dA}}{{dG}} = \frac{{\left[ {\left( {\theta + t} \right){f_{ee}} - v''} \right]S''}}{{\left| H \right|}} > 0. \end{equation*}

Intuitively, an increase in the incentive rate *t* leads to an increase in both effort and the amount of resources dedicated to nutrition (11–12). Notably, these increases are greater when *f_eA_* is larger. Naturally, the larger that *f_eA_* is, the larger is the marginal productivity of either *e* or *A* when the other input increases, accentuating the effect of increasing the incentive rate.

An increase in discretionary resources also raises both effort and resources devoted to nutrition (13–14). Note in (13) that if the marginal productivity of effort were independent of the level of *A*—that is, if *f_eA_* = 0, then changes in discretionary resources would not influence effort. This is not a general property, but rather a result of our simplifying assumption that $v$(*e*) and *S*(*G* − *A*) are additive in the utility function, so *e* and *A* only interact through the production function.

An important result is also that 0 < *dA*/*dG* < 1. This means that an increase in *G* translates into a positive but smaller increase in *A*, implying that the full increase in *G* is not entirely allocated to *A*, but rather a share is invested in non-nutritional activities. This is clear from (7): if *G* and *A* increased by the same amount, then the term *S*΄(*G* − *A*) would not change—and hence could not be equal to (θ + *t*)*f_A_*(*e*, *A*).

From first order conditions (11–14), it then follows that

(15) \begin{equation*} \frac{{\ dH}}{{dt}} = {f_e}\frac{{de}}{{dt}} + {f_A}\frac{{dA}}{{dt}} > 0, \end{equation*}

(16) \begin{equation*} \frac{{\ dH}}{{dG}} = {f_e}\frac{{de}}{{dG}} + {f_A}\frac{{dA}}{{dG}} > 0. \end{equation*}

To understand the conditions under which incentives and resources are complements or substitutes, we must compute the cross-partial derivatives of *e*,  *A*,  and *H* with respect to *t* and *G*. Note that because the first order comparative statics (11–14) depend on the second derivatives of $v$(*e*)*, S*(*G* − *A*), and *f*(*e, A*), the cross-partial derivatives will necessarily depend on the third order derivatives of $v$(*e*), *S*(*G* − *A*), and *f*(*e, A*). In order to gain insight, we make the simplifying assumption that the third order derivatives of the production function are null (i.e., that the production function is quadratic) while leaving $v$΄΄΄ and *S*΄΄΄ unrestricted.^10^

Using the chain rule on *H* = *f*(*e*,  *A*), the cross-partial derivative of *H* with respect to *t* and *G* is

(17) \begin{eqnarray*} \frac{{dH}}{{dtdG}} &=& \left[ {{f_{ee}}\frac{{de}}{{dG}}\frac{{de}}{{dt}} + {f_{AA}}\frac{{dA}}{{dG}}\frac{{dA}}{{dt}}} \right] + {f_{Ae}}\left[ {\frac{{de}}{{dG}}\frac{{dA}}{{dt}} + \frac{{de}}{{dt}}\frac{{dA}}{{dG}}} \right]\nonumber\\ &&+\, {f_e}\frac{{de}}{{dtdG}} + {f_A}\frac{{dA}}{{dtdG}}, \end{eqnarray*}

which could be positive (implying that *t* and *G* are complements) or negative (implying that they are substitutes) because the first term in brackets is negative, the second term is positive (and its size crucially depends on *f_eA_*), and the third and fourth could be positive or negative (as shown in Online Appendix B, which provides expressions for $de/dtdG$ and $dA/dtdG$). Because the sign of $dH/dtdG$ cannot be determined *a priori*, we discuss in what follows how its sign depends on the sign and size of key derivatives: *S*΄΄΄, $v$΄΄΄,  *f_eA_*.

A key determinant of $dH/dtdG$ is *f_eA_*, how much the productivity of effort increases when *A* increases. A larger *f_eA_* favors complementarity between *t* and *G* ($dH/dtdG$ > 0). Intuitively, larger values of *G* imply larger values of *A*, leading to effort being more productive (*f_eA_* > 0), and hence a larger response to the incentive.^11^ Mathematically, *f_eA_* multiplies the second term in (17), which is positive, and also enters into the formulae for *de*/*dtdG* and *dA*/*dtdG*.

The third derivative of $v$(*e*), $v$΄΄΄, defines whether the marginal cost of effort, $v$΄, is convex ($v$΄΄΄> 0) or concave ($v$΄΄΄< 0). A convex (concave) marginal cost of effort favor substitution (complementarity). To understand the intuition, assume that the marginal cost of effort is concave, that is, $v$΄΄΄< 0 (i.e., $v$(*e*) = *e*^α^, 2 < α < 3), and consider the following approximation:

(18) \begin{eqnarray*} \frac{{de}}{{dtdG}} &\approx& \frac{{\frac{{de\left( {G = {G^h},t} \right)}}{{dt}} - \frac{{de\left( {G = {G^l},t} \right)}}{{dt}}}}{{{G^h} - {G^l}}},\ \ \ \ {G^h} > {G^l}. \end{eqnarray*}

Note that $de/dG$ > 0,  so effort is greater for *G^h^* than for *G^l^*. Moreover, the concavity of the marginal cost of effort implies that, at higher levels of effort, the marginal cost of effort increases at a lower rate. Hence, the marginal cost of effort increases at a lower rate at *G^h^* than at *G^l^*. Hence, the response of effort to an increase in incentives, *de*(*G*,  *t*)/*dt*, that is, the terms in the numerator of (18), might be larger for *G^h^* than *G^l^* because the increase in the marginal cost of effort will be smaller.

A similar argument can be made to explain why *S*΄΄΄ > 0 is conducive to complementarity. Ultimately, both *e* and  *A* are inputs in the health production function, but $v$(*e*) is increasing in *e* whereas *S*(*G* − *A*) is decreasing in *A*. This explains why if $v$΄΄΄< 0 favors complementarity, *S*΄΄΄> 0 also does.^12^

The flexibility of the model means that our predictions depend on three key parameters. Having discussed the effect of each of them individually, Table 1 summarizes the necessary and sufficient conditions that the model provides.

**Table 1.** Summary of necessary and sufficient conditions implied by the model.

*S*΄΄΄ ≤ 0, and $v$΄΄΄ ≥ 0, and *f_eA_* = 0	*imply that*	$\frac{{dH}}{{dtdG}} < 0$
*S*΄΄΄ > 0, or $v$΄΄΄ < 0, or *f_eA_* > 0	*are necessary conditions for*	$\frac{{dH}}{{dtdG}} > 0$

## 3. Background

### 3.1. School-Based Nutrition Programs

School-based interventions are believed to be among the most cost-effective approaches for delivering health and nutrition services to children in developing countries (Bundy and Guyatt 1996; Jukes, Drake, and Bundy 2008; Orazem, Glewwe, and Patrinos 2008). Because schools are natural points of contact with school-aged children, they may provide a platform from which health and nutrition interventions can be delivered at relatively low cost (Bundy and Guyatt 1996; Bundy et al. 2006; Jukes, Drake, and Bundy 2008). Because of this, school-based health, nutrition and feeding programs are a ubiquitously central function of schools, particularly in developing countries.

In China, schools have the legal responsibility to promote the health of their students (Education Law of the Peoples Republic of China, 1995). Although school administrators are evaluated as part of the cadre evaluation system (*ganbu kaohe zhidu*)—a system for evaluating public officials and servants in China (Whiting 2004)—measures of child health are not typically included as criteria for evaluation.

### 3.2. The Causes and Consequences of Anemia

Our study examines school-based programs to reduce anemia. Anemia is estimated to affect nearly one quarter of all school-aged children worldwide (World Health Organization 2001). Although there are many causes of anemia (including a variety of genetic disorders and infections as well as nutritional deficiencies), iron deficiency accounts for about 50% of cases globally (Balarajan et al. 2011; Pasricha et al. 2013)^13^—and 85%–95% of cases in China (Du et al. 2000).

The consequences of iron deficiency—with or without anemia—can be substantial, particularly for children at critical stages of development. A large literature links iron deficiency to fatigue and reduced work capacity among adolescents and adults, impaired cognition and cognitive development among children, and reduced immune response for all age groups (Thomas et al. 2006; World Health Organization 2001; Yip 2001; Balarajan et al. 2011). School-aged children with anemia (the focus of our study) have also been shown to have inferior educational outcomes (grades, attendance, and school attainment—Taras 2005; Nokes, van den Bosch, and Bundy 1998).

### 3.3. Biomedical Strategies for Reducing Anemia

Increasing iron consumption can effectively prevent iron deficiency anemia. Worldwide, fortifying staple foods with iron has historically been an effective approach to addressing micronutrient deficiencies (Allen et al. 2006). Fortification is an attractive strategy because it requires little behavior change and because it can be implemented on a large scale. However, fortification of staple foods may be ineffective in areas like Northwest China in which households grow and consume their own food (Allen et al. 2006).

An alternative approach is to increase the consumption of naturally iron-rich foods and those that promote iron absorption during digestion. Animal sources (including red meats, fish, and poultry) provide *heme* iron, which is more easily absorbed during digestion; plant sources (including green, leafy vegetables) provide *nonheme* iron, which is less readily absorbed—but can be promoted by consumption of vitamin C (and inhibited by consumption of milk and other calcium-rich products).

Finally, a third approach is the delivery of micronutrient supplements (e.g., vitamins) containing iron. To be effective, however, regular consumption over several few months is necessary—and so inadequate compliance may render supplementation ineffective (Bobonis et al. 2006; Bhutta et al. 2013; Pasricha et al. 2013; Martorell et al. 2015).^14^

## 4. The Experiment

### 4.1. Sampling

**Figure 1.**
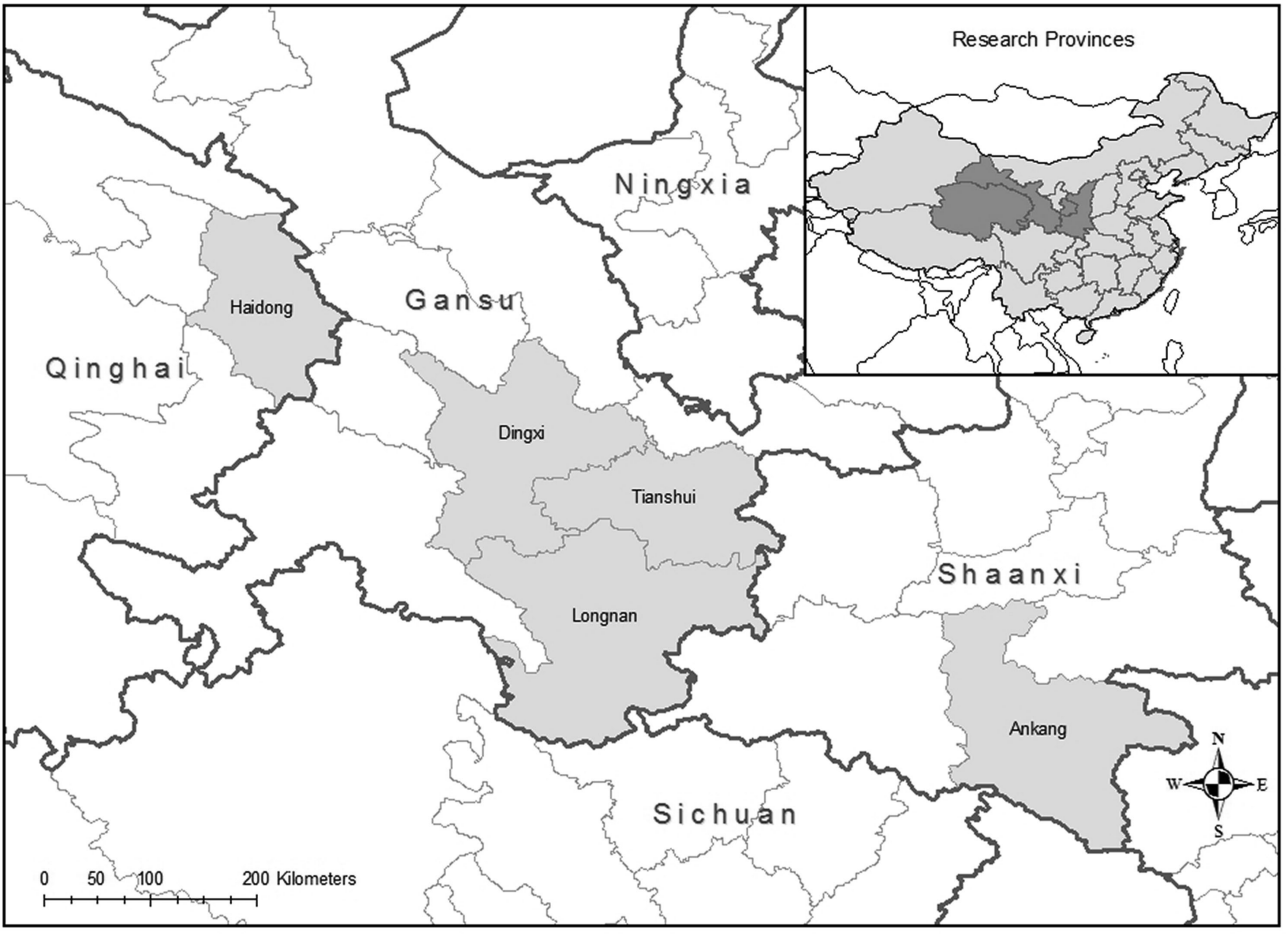
Study regions.

To draw our study sample, we began with all 36 counties officially designated by the Chinese government as “poverty counties” in five regions (prefectures) in western China (Haidong in Qinghai Province, Dingxi, Tianshui, and Longnan in Gansu Province, and Ankang in Shaanxi Province—see Figure 1). In August 2011, we conducted a canvass survey in each county to construct a list of all rural primary schools and the number of students enrolled in each. Restricting our sampling frame to primary schools with 150–300 students total,^15^ we randomly selected 170 of 1410 eligible schools for inclusion in our study (and limited our selection to one school per township^16^). Our sample size was based on power calculations conducted using data from primary schools in the same region of China (Miller et al. 2012).^17^

Within study schools, we randomly sampled 50 fourth and fifth grade students from each school. In China, fourth and fifth grade students are typically 10–11 years old, and we chose these grades to select students whom we considered sufficiently old to provide meaningful survey responses—but also sufficiently young to be generally prepubescent (given the independent effect of menarche on hemoglobin concentration). We also conducted physical exams and collected data from students from other grades at baseline to obfuscate our focus on fourth and fifth graders.

### 4.2. Data Collection

We conducted our baseline survey in September 2011 and our follow-up survey in May 2012 (at the beginning and end of the 2011–2012 academic year), collecting detailed information on students, households, school administrators, and schools.

#### Student Surveys.

We interviewed all sampled students at their school, collecting information on student background, health behaviors related to anemia, school activities, and general health. To collect information on school and home feeding practices, students were also given standard food frequency questionnaires to record information about food consumption at school and at home over the past week.^18^

We also measured student blood hemoglobin (Hb) concentration at the time of the student survey. Nurses from the Medical School of Xi’an Jiaotong University accompanied study enumerators, collecting finger-prick blood samples to analyze on-site (at schools) using HemoCue Hb 201+ assessment systems.

#### Household Surveys.

For each sampled student, we also collected information on students’ households using forms completed by parents.^19^ Specifically, these surveys collected information about interactions between parents and the school, household income and assets, health-related expenditures, expenditures on food and information on other household members, focusing on household characteristics that students would be unlikely to know themselves.

#### School Administrator Surveys.

We interviewed school administrators (bureaucrats) at three different points in time: before and after school administrators were told about the incentive contract and block grant to which they were assigned and again at endline. At baseline, school administrators provided information about their background, job history, salary, and compensation as well as perceptions of professional responsibilities and anemia knowledge. Using scales adapted from Grant (2008), we also measured the intrinsic and prosocial motivation of administrators. Following their participation in the training session on anemia (conducted 3 weeks after the baseline survey) administrators were given a second short survey to measure their understanding of the training material.

#### School Surveys.

Finally, we collected basic information from schools (about enrollment, staffing, facilities, finances, and meal provision) and teachers (about teacher characteristics, communication with parents, and teaching practices).

### 4.3. Experimental Design

**Figure 2.**
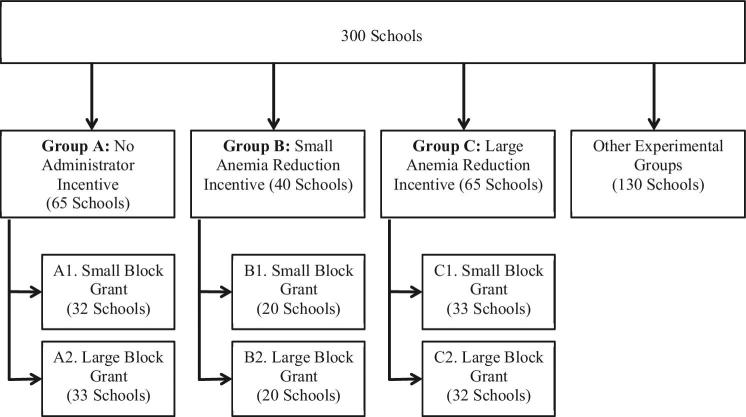
Experimental design.

**Figure 3.**
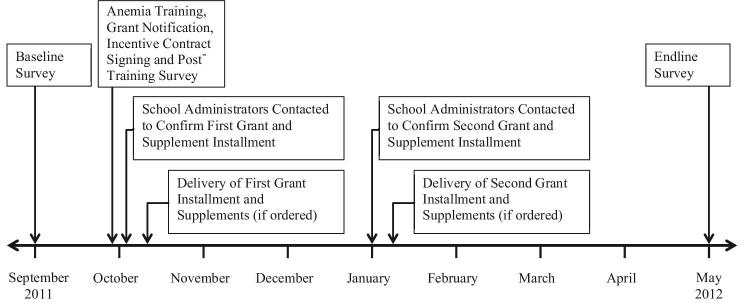
Data collection and intervention timeline.

We designed our study as a cluster-randomized trial using a 3 × 2 crosscutting design (Figure 2). After conducting our baseline survey, we provided all school administrators with information about anemia (which included presentations and a video presentation by a Chinese nutrition specialist, see script in Online Appendix C), and schools were randomly assigned one of six experimental cells (see Figure 3 for the study timeline). The first three paths of Figure 2 show randomly-assigned incentive groups: a group without incentives (group A), a “small” incentive group (group B), and a “large” incentive group (group C). Across these arms are two orthogonally assigned block grant groups: a “small” block grant group (group 1) and a “large” block grant group (group 2). The reference group in our six-cell design is the default policy (education about anemia coupled with a modest resource transfer and no incentives, group A1).^20^

To improve power, we used a stratified randomization procedure. Specifically, using joint quintiles of the baseline distribution of school-level hemoglobin concentration and combined standardized math and Chinese exam scores—yielding 25 strata, we randomized cell assignment within each stratum. Stratification improves power by ensuring balance on these covariates between experimental groups. Our analysis takes this randomization procedure into account, conditioning on stratum fixed effects (Bruhn and McKenzie 2009).

#### Incentives for Anemia Reduction.

In the large incentive arm (65 schools, group C in Figure 2), we offered school administrators financial incentives to be paid as private income according to the net reduction in number of students identified as anemic between the beginning and end of the school year. The specific structure of the large incentive contract was

$$\begin{equation*}
P = \left\{ {\begin{array}{@{}*{1}{c}@{}} {125\ {{\rm RMB}}\ \times \ \left( {{N_b} - {N_e}} \right)\ \ \ \ \ \ \ \ \ \ {\rm if}\ \left( {{N_b} - {N_e}} \right)\ > \ 0}\\ {0\ \ \ \ \ \ \ \ \ \ \ \ \ \ \ \ \ \ \ \ \ \ \ \ \ \ \ \ \ \ \ \ \ \ \ \ \ \ \ \ \ \ \ \ \ \ {\rm otherwise}} \end{array}} \right.,
\end{equation*}$$

where *N_b_* is the number of students found to be anemic at baseline and *N_e_* is the number of who were anemic at the time of the endline survey.^21^ Based on an earlier study (Miller et al. 2012), the contract increment (125 yuan (RMB), or about $19.40^22^) per student reduction was chosen to provide roughly two months of a school administrator's annual salary for a feasible reduction in anemia given previous studies (a reduction of about 50%).^23^ Actual payouts for school administrators with the large incentive and small block were ultimately 3,303 yuan (or about $516)—approximately two month's base pay for school administrators in this region. We did not reveal the identity of students who were anemic at baseline to administrators (and when we asked teachers to identify students who were anemic at endline, they were unable to do so).^24^

The small incentive arm (40 schools, group B in Figure 2) was identical to the large incentive arm except that the magnitude of the incremental incentive was ten times smaller (12.5 RMB, or about $1.95 per student reduction in anemia between baseline and follow-up in our sample). This magnitude of this incentive provides roughly 0.2 additional months of annual salary for the same feasible reduction in anemia given previous studies.

At the time that school administrators signed incentive contracts, they were told the (implied) number of anemic students in their schools (the identity of anemic children was not revealed).^25^ Contracts were written using official letterhead of the Chinese Academy of Sciences (a government agency) and counter-signed by the deputy director of the implementing research center (school administrators signed two copies of the contract, one of which they kept). Note that all interventions were implemented in partnership with local education bureaus, signifying to school administrators that the project was sanctioned by local governments.

#### Block Grants.

**Figure 4.**
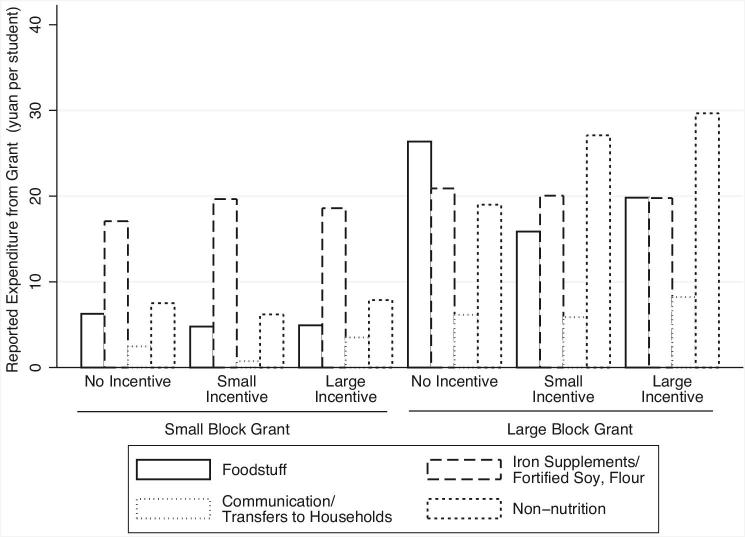
Reported use of block grants by category. Figure shows mean values of reported use of block grants by experimental group from the endline survey. Expenditure amounts are per student.

The small block grant (group 1 in Figure 2) was 0.3 RMB ($0.05) per student per day (85 schools), which we calculated to be adequate for school administrators to purchase vitamins for each student to take daily. The large block grant (group 2 in Figure 2) was 0.7 RMB ($0.11) per student per day (85 schools). In total, small block grant schools received 7,452 yuan ($1164) on average and large block grant schools received 17,388 yuan ($2717). These grants were given to schools in two installments, once at the beginning of the program and another approximately half way through the school year.^26^ Although funds were given in the context of the nutrition program roll-out, administrators were explicitly told that they were free to allocate these at their discretion to other school functions benefitting students—whether this be for educational goods, health specific goods, or general school supplies.^27^ Indeed, Figure 4 shows that administrators used a substantial share of their grants for activities unrelated to nutrition.

#### Health Education.

Because knowledge about anemia in our study areas was poor, prior to revealing treatment assignment, we provided health education about nutrition and anemia to all school administrators in our study (see Online Appendix C). Our health education materials were based on published, peer-reviewed studies and specifically included information about: (1) the prevalence and causes of anemia, (2) the consequences of anemia (including its effect on cognitive development and academic performance), and (3) efficacious nutritional approaches to reduce anemia (increasing dietary intake of iron-rich foods, nutritional supplementation with iron fortified soy and flour or with supplements, etc.).

### 4.4. Balance and Attrition

Summary statistics and tests for balance across study arms are shown in Table 2.^28^ Panel A shows student level characteristics (*N* = 2051), panel B shows characteristics of schools (*N* = 167), and panel C shows characteristics of school administrators (*N* = 167).^29^ The first two columns of the table give the mean and standard deviation of each variable in the comparison (small block grant, no incentive) group. Columns (3)–(7) show coefficients on treatment variables and interactions estimated using equation (19), controlling only for randomization strata fixed effects. The final column shows the *p*-value from a test that the coefficients are jointly zero for each characteristic. Only 4 of the 75 tests are significant at the 10% level, and a test for joint equality is rejected at the 10% level for only one characteristic (the number of times meat was consumed in the past week). Joint tests for all 15 characteristics reveal no significant differences.^30^

**Table 2.** Descriptive statistics and balance check.

		No incentive, small grant group		Coefficient (standard error) on						
		Mean	SD	Small incentive	Large incentive	Large grant	(Small-incentive) × (large grant)	(Large incentive) × (large grant)	Joint Test *p*-value: all coefficients = 0	Observations
		(1)	(2)	(3)	(4)	(5)	(6)	(7)	(8)	(9)
*Panel A: Child characteristics*										
(1)	Hemoglobin concentration (g/L)	118.446	7.541	$-$0.965	$-$1.525	$-$0.653	2.868	1.479	0.420	2051
				(1.326)	(1.163)	(1.438)	(1.959)	(1.761)		
(2)	Age (years)	10.514	1.153	0.046	0.077	0.113	$-$0.002	$-$0.070	0.914	2051
				(0.166)	(0.125)	(0.134)	(0.242)	(0.189)		
(3)	5th Grade (0/1)	0.468	–	0.055*	$-$0.002	$-$0.003	$-$0.106**	0.016	0.177	2051
				(0.032)	(0.029)	(0.031)	(0.050)	(0.042)		
(4)	Female (0/1)	0.530	–	0.003	$-$0.021	$-$0.009	0.001	0.044	0.945	2051
				(0.044)	(0.035)	(0.039)	(0.060)	(0.052)		
(5)	Times consumed meat in past week (incl. chicken, pork, beef, lamb)	3.922	4.145	$-$0.534	$-$1.293***	$-$0.352	$-$0.039	0.909	0.091*	2051
				(0.618)	(0.453)	(0.709)	(0.888)	(0.790)		
*Panel B: School characteristics*										
(6)	Number of students	203.733	55.788	2.424	7.060	$-$1.925	21.948	9.631	0.725	167
				(16.959)	(14.194)	(15.304)	(25.245)	(20.780)		
(7)	Has kitchen (0/1)	0.067	–	0.135	0.068	0.054	$-$0.071	$-$0.052	0.732	167
				(0.099)	(0.077)	(0.085)	(0.161)	(0.120)		
(8)	Student–teacher ratio	16.192	4.356	2.859**	1.190	0.019	$-$1.804	0.866	0.185	167
				(1.377)	(1.216)	(1.182)	(1.928)	(1.678)		
(9)	Time to furthest village served (min)	61.167	37.570	12.294	$-$2.256	4.020	$-$7.468	4.605	0.918	167
				(13.474)	(11.962)	(12.520)	(21.139)	(17.681)		
(10)	Percent boarding students (%)	4.277	9.493	2.228	0.756	1.310	$-$0.757	$-$1.804	0.985	167
				(3.976)	(2.899)	(3.400)	(6.204)	(5.107)		
*Panel C: School administrator characteristics*										
(11)	Male (0/1)	0.967	–	$-$0.015	0.028	0.038	0.014	$-$0.070	0.606	167
				(0.051)	(0.032)	(0.034)	(0.053)	(0.046)		
										
(12)	Age (years)	39.567	7.398	1.550	1.299	1.599	$-$4.730	0.090	0.383	167
				(2.112)	(1.837)	(1.882)	(3.022)	(2.601)		
(13)	Higher education degree (0/1)	0.900	–	0.018	$-$0.007	$-$0.107	0.032	$-$0.007	0.558	167
				(0.092)	(0.081)	(0.093)	(0.136)	(0.126)		
(14)	Experience (years)	8.333	6.227	$-$0.194	1.124	0.898	$-$2.761	$-$0.165	0.137	167
				(1.531)	(1.786)	(1.630)	(2.210)	(2.577)		
(15)	Monthly base salary (yuan)	1855.067	706.106	$-$57.049	$-$110.575	$-$36.880	$-$312.491	$-$35.944	0.602	167
				(196.310)	(178.286)	(182.302)	(305.716)	(247.052)		

Notes: Table uses sample of children testing anemic at baseline. Children are considered anemic if they have an altitude-adjusted hemoglobin concentration below 120 g/L (per WHO guidelines). The first and second columns show the mean and standard deviation in the comparison (small grant, no incentives) group. Columns (3) through (7) show coefficients and standard errors from a regression of each characteristic on indicators for incentive and large grant treatment group indicators and there interactions, controlling for randomization strata. Column (8) shows the *p*-value from a test that coefficients are jointly zero. All tests account for clustering at the school level. *Significant at 10%; **significant at 5%; ***significant at 1%. Source: Baseline survey.

The overall attrition rate between baseline and endline surveys was 6.2% in our sample of children anemic at baseline (5% for the full sample). Defining attrition as a missing hemoglobin measurement at endline for students with a baseline measurement, Online Appendix Table A.2 shows that there were no meaningful differences in attrition across treatment groups (columns (1) and (2)). Analyzing the correlates of a missing household survey at endline conditional on a child not dropping out, Online Appendix Table A.2 also shows that neither the treatment indicators nor other covariates are significantly correlated with a missing household survey form.

### 4.5. Empirical Strategy

Given random assignment of schools to treatment cells shown in Figure 2, comparisons of outcome variable means across treatment groups provide unbiased estimates of the effect of each experimental treatment. However, to increase power (and to account for our stratified randomization procedure), we condition our estimates on a set of covariates used in power calculations. With few exceptions, all of the analyses presented (including outcome variables, regression specifications, and hypotheses tested) were prespecified in a preanalysis plan written and filed before endline data were available for analysis.^31^ In reporting results in what follows, we explicitly note analyses that deviate from the preanalysis plan.

As specified in advance, we use ordinary least-squares (OLS) regression to estimate the effect of cell assignment on child-level outcomes with the following specification:

(19) \begin{eqnarray*} {Y_{i,j}} &=& \alpha + {\beta _1}S{I_j} + {\beta _2}L{I_j} + {\beta _3}L{G_j} + {\beta _4}( {S{I_j}}) \times ( {L{G_j}}) \nonumber\\ &&+\,{\beta _5}( {L{I_j}}) \times ( {L{G_j}})+ X_{i,j}^{'}\gamma + {\varepsilon _{i,j}}, \end{eqnarray*}

where *Y*_*i*, *j*_ is the outcome for child i in school j; *SI_j_* is a dummy that equals 1 if the administrator in school *j* was assigned to receive a small incentive contract and 0 otherwise; *LI_j_* is equal to 1 if the administrator in school *j* was assigned to receive a large anemia reduction incentive contract; *LG_j_* is equal to 1 if the school received a large block grant; *X*_*i*, *j*_ is a vector of child controls (age, class-year, and gender, and baseline value of the outcome variable), school controls (number of students, student-teacher ratio, whether the school has a kitchen, proportion of boarding students, and distance to the farthest village in the school's catchment area); and dummy variables for counties and randomization strata. We adjusted our standard errors for clustering at the school level using Liang–Zeger clustered standard errors.

In addition to estimating effects on our two primary outcomes (hemoglobin concentration and a dichotomous indicator for anemia status), we use the same specification to estimate effects on secondary outcomes to examine the behavioral mechanisms underlying changes in primary outcomes. For these secondary outcomes, we focus our analysis on summary indices constructed using groups of closely-related outcome variables (as we specified in advance). To construct these indices, we used the GLS weighting procedure described by Anderson (2008). For each individual, we constructed a variable ${\bar{s}_{ij}}$ as the weighted average of *k* normalized outcome variables in group ($y_{ijk}$). The weight placed on each outcome variable is the sum of its row entries in the inverted covariance matrix for group *j* such that

$$\begin{equation*}
{\bar{s}_{ij}} = {\left( {{\boldsymbol{1}}{\rm{^{\prime}}}{{\widehat {{{{\bf \sum }}_j}}}^{ - 1}}1} \right)^{ - 1}}\left( {{\boldsymbol{1}}{\rm{^{\prime}}}{{\widehat {{{{\bf \sum }}_j}}}^{ - 1}}{{\boldsymbol{y}}_{ij}}} \right),
\end{equation*}$$

where 1 is a column vector of 1 s, ${\widehat {{{{\bf \sum }}_j}}^{ - 1}}$ is the inverted covariance matrix, and ***y**_ij_* is a column vector of all outcomes for individual *i* in group *j*. In addition to reducing the number of tests required, this weighting procedure can improve efficiency by placing less weight on outcomes that are highly correlated and more weight on those less correlated. The summary index variable can also be created for individuals with a subset of missing outcomes (these outcomes simply receive less weight in the construction of the index). Although we emphasize these indices in our discussion, we also report estimates for each individual index component in Online Appendix Tables A.5–A.8.

A note on correcting for multiple comparisons is also warranted. For our primary estimates, we test eight null hypotheses: five for treatment main effects and their interactions (shown in equation (19)) and three additional ones—that the small and large incentives have the same average effect (β_1_ = β_2_), that the large incentive and the large block grant have the same average effect (β_2_ = β_3_), and that the average effect of the large incentive in presence of a large grant is zero (β_2_ + β_5_ = 0).^32^ We therefore adjust our *p*-values to control the family wise error rate (FWER), or the probability of at least one Type I error. Specifically, we use the free step-down resampling method of Westfall and Young (1993). This procedure accounts for the dependency of the data, and is therefore more powerful than procedures that do not (e.g. a Bonferroni correction ). For secondary outcomes, we adjust our *p*-values according to the total number of tests within a family of outcomes (the number of outcomes in the family times five—the number of treatment coefficients in each regression).

## 5. Results: Childhood Anemia and Underlying Behavioral Responses

In this section, we first present results obtained by estimating equation (19) for anemia status and hemoglobin concentration, and in Section 5.2, we then investigate the underlying behavioral responses that may have produced them. Following our preanalysis plan, we emphasize estimates from our sub-sample of children who were anemic at baseline. In the Online Appendix Tables we report results for the full sample of children receiving hemoglobin tests (Online Appendix Tables A.3, A.4, A.6, and A.8).

### 5.1. Childhood Anemia

The first five rows of Table 3 report estimates for each treatment and their interactions (and the seventh row reports comparison group means for the no incentive, small grant group at endline). For each estimate, we report the regression coefficient, the standard error and corresponding *p*-value, and the *p*-value adjusted for multiple hypotheses testing.^33^

**Table 3.** Impacts of school administrator anemia reduction incentives and block grant size on student hemoglobin concentration and anemia prevalence.

		Anemic at endline (Hb < 120 g/L)	Hemoglobin concentration (g/L)
Dependent variable		(1)	(2)
*Panel A: Impacts relative to comparison (no incentive, small grant) group*			
(1)	β_1_: small incentive	$-$0.012	$-$0.387
		(0.040)	(1.101)
		[0.771]	[0.726]
		{0.972}	{0.792}
(2)	β_2_: large incentive	$-$0.138*	2.567
		(0.039)	(1.044)
		[0.001]	[0.015]
		{0.064}	{0.285}
(3)	β_3_: large grant	$-$0.145**	4.205**
		(0.038)	(1.123)
		[<0.001]	[<0.001]
		{0.047}	{0.045}
(4)	β_4_: (small incentive) × (large grant)	$-$0.042	1.445
		(0.056)	(1.541)
		[0.453]	[0.350]
		{0.888}	{0.664}
(5)	β_5_: (large incentive) × (large grant)	0.196*	$-$4.580
		(0.058)	(1.586)
		[<0.001]	[0.004]
		{0.072}	{0.173}
(6)	Observations	1923	1923
(7)	Mean in no incentive, small grant group	0.364	129.901
*Panel B: *p*-values of Additional Comparisons*			
(8)	β_1_–β_2_: effect of large incentive versus effect of small incentive given small grant	[0.002]	[0.014]
		{0.089}	{0.285}
(9)	β_2_–β_3_: effect of large incentive given small grant versus effect of increasing grant amount	[0.854]	[0.169]
		{0.972}	{0.597}
(10)	β_2_ + β_5_: effect of large incentive given large grant	[0.141]	[0.080]
		{0.650}	{0.511}

Notes: Table uses sample of children testing anemic at baseline. Children are considered anemic if they have an altitude-adjusted hemoglobin concentration below 120 g/L (per WHO guidelines). Rows (1)–(5) in panel A show estimated coefficients for treatment group indicators and interactions obtained by estimating equation (19) (controlling for baseline hemoglobin concentration, student age, student grade, student sex, number of students in the school, whether the school has a canteen, student teacher ratio, distance to the furthest village served, percent of boarding students, whether the school has implemented the “Free Lunch” policy, county dummy variables, and dummy variables for randomization strata). Standard errors are shown in parentheses, unadjusted *p*-values are shown in square brackets and *p*-values adjusted for multiple inference are shown in curly brackets. Adjusted *p*-values were constructed using the free step-down resampling method of Westfall and Young (1993) with 10,000 iterations. Panel B shows unadjusted and adjusted *p* values from tests of linear combinations of coefficients in panel A. *Significant at 10%; **significant at 5%; ***significant at 1% based on adjusted *p* values.

Result 1. (Large Incentives). First, we find that the large incentive significantly reduced the probability of anemia at endline in schools receiving a small grant. Specifically, the large incentive was associated with a 14 percentage point reduction in anemia (Table 3, row 2, column (1); unadjusted *p*-value = 0.001, adjusted *p*-value = 0.064), implying a 37.9% reduction relative to the comparison group (small grant, no incentive schools) at endline. The corresponding increase in hemoglobin was about 2.6 g/L (Table 3, row 2, column (2); unadjusted *p*-value = 0.015, adjusted *p*-value = 0.285). These empirical findings agree with our model's prediction derived in equations (11) and (12).

**Figure 5.**
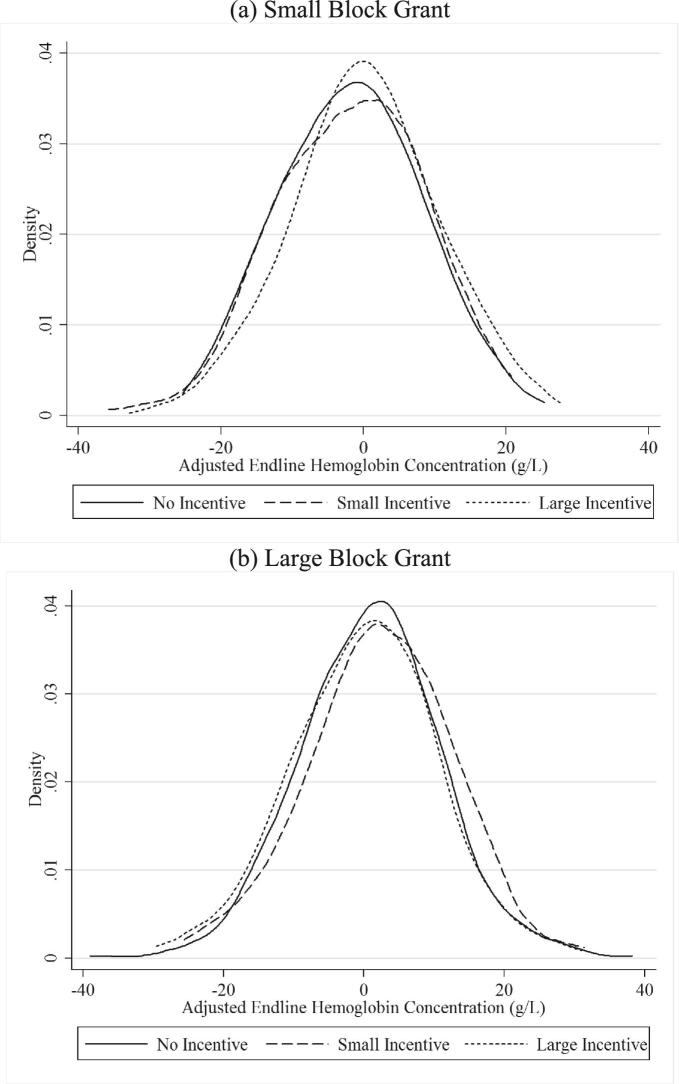
Distributions of hemoglobin concentration. (a) and (b) plot the distributions of student level hemoglobin concentrations (in g/L) at endline across incentive treatment groups separately by small and large block grant groups. Endline Hb concentrations are adjusted for prespecified baseline control variables. Kolmogrov–Smirnov *p* values for small anemia incentive versus no incentive are in 0.93 panel (a) and 0.12 in panel (b). For large anemia versus no incentive these are in 0.02 panel (a) and 0.24 in panel (b).

Because our incentives rewarded anemia reduction (and not hemoglobin levels per se) and anemia status reflects shifts in the distribution of altitude adjusted hemoglobin concentrations across the 120 g/L threshold, Figure 5a plots the distribution of endline hemoglobin concentrations (adjusted for covariates included in equation (19)) by study arm among children who were anemic at baseline. The distribution for the large incentive group is shifted to the right of the control group distribution (Kolmogorov–Smirov test *p*-value = 0.02). This relative shift in mass is greater in the left tail of the distribution, implying that the large incentive reduced the share of children falling below the anemia threshold.

Result 2. (Small Incentives). Second, in contrast, the small incentive had no detectable effect on the probability of anemia at endline (Table 3, row 1, column (1)). Comparing the estimates for small and large incentives (β_1_ =  β_2_  in equation (19)),  we also reject the null hypothesis that the two estimates are equal (Table 3, row 8, column (1); adjusted *p*-value = 0.089). Taken together, the estimates for the small and large incentives suggest that the price effect of incentives is meaningful independent of information conveyed by the presence of an incentive contract (Gneezy and Rustichini 2000). Figure 5a shows that the shift in the hemoglobin distribution for the small incentive arm relative to the control group arm is smaller—particularly in the left tail of the distribution.

An important question in the literature on financial incentives is whether or not they crowd-out intrinsic or prosocial motivation (Deci and Ryan 1985; Gneezy and Rustichini 2000; Fehr and Falk 2002; Francois and Vlassopoulos 2008; Gneezy, Meier, and Rey-Biel 2011; Kamenica 2012). We find that the effects of the small incentive on anemia was significantly more positive amongst school administrators who score higher at baseline on a prosociality scale (adapted from Grant 2008) (Online Appendix Table A.10, row 1, columns (1)–(3); adjusted *p*-value = 0.038). We also find a similar effect for intrinsic motivation (also adapted from Grant 2008), but the difference in effects is not statistically significant (Online Appendix Table A.10, row 4, columns (1)–(3); adjusted *p*-value = 0.570). However, the effect of the large incentive is not heterogeneous by prosocial or intrinsic motivation (the coefficients are close to zero and not statistically significant), implying that if monetary incentives are large, crowding-out of prosocial motivation may be overcome by extrinsic motivation provided by incentives.

Result 3. (Large Block Grants). Third, in the absence of any explicit incentive, the large block grant alone reduced the probability of student anemia at endline (an unambiguous prediction of our model, as equations (13), (14), and (16) show). Specifically, Table 3 (column (1), row 3) shows that the large block grant was associated with a 14.5 percentage point reduction in anemia (adjusted *p*-value = 0.047), implying a 39.8% reduction relative to the comparison group at endline. This reduction is very similar to the effect of the large incentive (−0.145 vs. −0.138), but the average increase in hemoglobin concentration is larger (4.205 vs. 2.567), although not statistically so (Table 3, row 9, column (2); adjusted *p*-value = 0.597).

Result 4. (Interactions between Incentives and Grants). Whether or not incentives and unrestricted grants are complements or substitutes is an empirical issue. The model in Section 2 makes clear that both complements or substitutes are possible depending on cross partial derivatives of the hemoglobin production function as well as the curvature of the marginal cost of effort and the marginal utility that the school administrator obtains from non-nutritional activities. We do not find evidence of complementarity—and notably, incentives and block grants can be strong substitutes if the incentives are sufficiently large.

Table 3 shows that the interaction between the large incentive and the large block grant (β_5_ in equation (19); Table 3, column (1), row 5) is positive and statistically significant (adjusted *p*-value = 0.072). Moreover, the magnitude of substitution implies that the large incentive and the large block grant fully crowd each other out: the marginal effect of the large incentive given the large block grant in column (1) (β_2_ + β_5_ =  0.058) is not statistically different from zero (adjusted *p*-value = 0.65) for the probability of anemia.^34^ Although this point estimate is positive, we are not able to rule out a negative effect of the large incentive given a large block grant on anemia as predicted by equation (15) of the model in Section 2. Adding coefficients, the estimated total effect of the large incentive and large grant on anemia is −0.087 (adjusted *p*-value: 0.177).

Given decreasing marginal returns to inputs in the reduction of anemia, a natural question arising from these results is if substitution between incentives and resources is due to (i) the biological relationship between inputs and anemia (i.e., although more inputs are used, there is no effect on anemia because of biological constraints) or (ii) conscious decisions by administrators. Our results for input use in Section 5.2 are consistent with the latter interpretation (we find direct evidence of substitution in input use). (We also note that efficacy trials of iron supplementation suggest that much larger reductions in anemia are biologically possible (Gera et al. 2007).) Given that we find similar results for input use in the full sample (Online Appendix Table A.4), differences in the effects of incentives and resources on anemia rates between the sample of children anemic at baseline (Table 3) and the full sample (Online Appendix Table A.3) are likely explained by decreasing marginal returns to inputs in the reduction of anemia rather than diminishing marginal returns to effort on the part of administrators.

### 5.2. Behavioral Responses Underlying Changes in Anemia

We next examine the underlying behavioral responses to our interventions that may have produced the changes in anemia described in Section 5.1. To do so, we focus on actions taken by administrators and subsequent responses among students and their parents—specifically, student consumption of iron-rich foods, direct iron supplementation, communication between parents and schools about anemia and its nutritional basis. For each family of outcome variables, we examine indices as described in Section 4.5.

#### Behavioral Responses Underlying Result 1: Large Incentives.

We first consider the behavioral responses underlying Result 1—that in the presence of the small block grant, the large incentive significantly reduced the probability of student anemia. The results in Table 4 suggest that the large incentive led administrators to increase vitamin supplementation and the provision of iron-rich foods (column (1), row 2; adjusted *p*-value 0.105). This increase in iron-rich foods seems driven largely by home consumption (row 2, column (5), adjusted *p*-value 0.090).^35^

**Table 4.** Child and household reported receipt of supplements and iron-rich food.

Dependent variable		Index: supplements and food	Subindex: supplements	Subindex: food	Subindex: food at school	Sub-index: food at home	Index: information	Subindex: information to students	Subindex: information to households
		(1)	(2)	(3)	(4)	(5)	(6)	(7)	(8)
(1)	β_1_: small incentive	0.059	0.138	−0.033	−0.08	0.072	0.027	0.062	0.045
		(0.047)	(0.084)	(0.044)	(0.055)	(0.061)	(0.075)	(0.126)	(0.084)
		[0.21]	[0.104]	[0.452]	[0.151]	[0.243]	[0.724]	[0.623]	[0.594]
		{0.331}	{0.234}	{0.56}	{0.555}	{0.403}	{0.935}	{0.911}	{0.690}
(2)	β_2_: large incentive	0.114	0.158	0.073	0.039	0.187*	0.070	0.111	0.130
		(0.045)	(0.080)	(0.038)	(0.048)	(0.064)	(0.072)	(0.101)	(0.084)
		[0.011]	[0.052]	[0.057]	[0.427]	[0.004]	[0.332]	[0.272]	[0.124]
		{0.105}	{0.234}	{0.339}	{0.601}	{0.090}	{0.800}	{0.796}	{0.430}
(3)	β_3_: large grant	0.190***	0.241*	0.138*	0.133	0.189	0.162	0.136	0.233
		(0.041)	(0.072)	(0.047)	(0.06)	(0.075)	(0.076)	(0.107)	(0.113)
		[<0.001]	[0.001]	[0.004]	[0.029]	[0.013]	[0.034]	[0.207]	[0.041]
		{0.004}	{0.051}	{0.092}	{0.27}	{0.126}	{0.326}	{0.768}	{0.352}
(4)	β_4_: (small incentive) × (large grant)	−0.200*	−0.269	−0.114	−0.105	−0.150	−0.037	0.190	−0.314
		(0.068)	(0.116)	(0.072)	(0.09)	(0.105)	(0.123)	(0.178)	(0.148)
		[0.004]	[0.021]	[0.116]	[0.243]	[0.157]	[0.766]	[0.288]	[0.036]
		{0.071}	{0.186}	{0.36}	{0.601}	{0.403}	{0.935}	{0.796}	{0.352}
(5)	β_5_: (large incentive) × (large grant)	−0.248**	−0.289	−0.212*	−0.209	−0.292	−0.117	−0.039	−0.356
		(0.064)	(0.106)	(0.067)	(0.079)	(0.110)	(0.108)	(0.151)	(0.146)
		[<0.001]	[0.007]	[0.002]	[0.009]	[0.009]	[0.282]	[0.797]	[0.016]
		{0.016}	{0.124}	{0.074}	{0.16}	{0.122}	{0.800}	{0.911}	{0.271}
(6)	Observations	1932	1932	1928	1920	1927	1932	1928	1548
(7)	Mean in no incentive, small grant group	0.031	−0.055	0.039	0.033	−0.053	−0.040	−0.017	−0.082

Notes: Table uses sample of children testing anemic at baseline. Children are considered anemic if they have an altitude-adjusted hemoglobin concentration below 120 g/L (per WHO guidelines). Rows (1)–(5) show estimated coefficients for treatment group indicators and interactions obtained by estimating equation (12) (controlling for the baseline value of the dependent variable, student age, student grade, student sex, number of students in the school, whether the school has a canteen, student teacher ratio, distance to the furthest village served, percent of boarding students, whether the school has implemented the “Free Lunch” policy, county dummy variables, and dummy variables for randomization strata). The dependent variable in each regression is a summary index constructed using the GLS weighting procedure in Anderson (2008). Estimates for the individual components of each index are shown in Online Appendix Tables A.5 and A.6. Standard errors are shown in parentheses, unadjusted *p* values are shown in square brackets and *p* values adjusted for multiple inference are shown in curly brackets. Adjusted *p* values were constructed using the free step-down resampling method of Westfall and Young (1993) with 10,000 iterations. *Significant at 10%; **significant at 5%; ***significant at 1% based on adjusted *p*-values.

An interesting issue is if the increase is vitamin supplementation and provision of iron-rich foods occurred because school administrators with large incentives spent the block grant differently—or instead because they exerted more effort. As Figure 4 shows, reported use of block grants for different types of nutrition interventions (vitamins, food, fortification), and other uses is similar for incentive and nonincentives schools receiving a small grant, suggesting that greater anemia reduction due to incentives is driven by effort rather than differential allocation of the block grant.

In exploring how administrators were able to increase child consumption of iron rich foods at home, we examine contact with parents. Row 2, column (8) of Table 4 reports a positive (but insignificant) increase in contact. However, Online Appendix Table A.7 shows that estimates for several components of this index appear meaningful and important, albeit insignificant at conventional levels using adjusted *p*-values (largely because of the large number of hypotheses being tested (11 × 5 = 55).^36^ These results are suggestive that the large incentive led administrators to engage more regularly with households—specifically about nutrition and anemia—which in turn appears to have improved children's diets at home.

The finding that administrators responded to large incentives by engaging with households is important for at least two related reasons. First, it demonstrates innovation and the use of local knowledge in response to performance incentives that reward outputs (health outcomes) as opposed to those that rigidly reward the use of prespecified inputs (such as vitamin consumption at school), as most performance incentives in the health sector do (Miller and Singer Babiarz 2014). Second, for outcomes jointly produced with beneficiary households (like good child nutrition), it demonstrates the potential of performance incentives that reward outputs to minimize offsetting compensatory behavior among beneficiaries (e.g. a common finding among studies of school lunch programs, ) (Jacoby 2002; Leonard 2003; Kazianga et al. 2009; Das et al. 2013).^37^

#### Behavioral Responses Underlying Result 2: Small Incentives.

Second, we study the behavioral responses underlying Result 2—that the small incentive did not reduce anemia prevalence. Table 4 (column (1)) shows that administrators with small incentives did not significantly increase the provision of supplements or food (row 1, columns (1)–(3)), nor did they increase their contact with households (column (8)) (Online Appendix Table A.7, row 1 also shows that none of the individual components of this index are statistically significant (even using unadjusted *p*-values).

#### Behavioral Responses Underlying Result 3: Large Block Grants.

Third, we examine behavioral responses to large block grants, which reduced the prevalence of student anemia. The large block grant significantly increased the provision of supplements and food (Table 4, row 3, column (1); adjusted *p*-value 0.004). This increase appears due to increases in both iron supplements (column (2), adjusted *p*-value 0.051) and iron-rich food (column (3), adjusted *p*-value 0.092).

Interestingly, the large block grant may have also increased school contact with parents—suggesting that administrators worked through households to reduce anemia without any explicit incentives to do so. Although the estimate for the index in Table 4 is not statistically significant (row 3, column (8)), some estimates for index components are larger than those for incentives. This may reflect intrinsic or prosocial motivation—or a sense of obligation or organizational mission (Ashraf, Bandiera, and Jack 2014). Furthermore, although the large block grant increased communication with households, the impact of block grants on food consumption at home is insignificant. We speculate that this could reflect less effort (relative to administrators with incentives) devoted to mitigating compensatory behavior by households in response to greater food provision at school (which seems to have increased, although not significantly, with large grants).

#### Behavioral Responses Underlying Result 4: Substitution between Large Incentives and Large Block Grants.

Finally, with the combination of large incentives and large block grants, we find direct evidence of crowding-out of inputs consistent with our anemia estimates in Table 3. Specifically, Table 4 shows that for vitamin supplementation and consumption of iron-rich foods (both at school and at home), estimates for the interaction between the large incentive and large block grant are negative, implying substitution (row 5). The interaction between the small incentive and large grant is also negative, but smaller in magnitude and only marginally significant. Overall, there is no evidence that resources and incentives are complements—and that at sufficiently high levels, they are substitutes.^38^

### 5.3. Comparative Cost-Effectiveness

Finally, we examine the comparative cost-effectiveness of each of our intervention combinations. In doing so, we consider both the subsample of children anemic at baseline and our full sample of children, and we present both “programmatic” cost-effectiveness (direct monetary program costs to the implementing organization) and social cost-effectiveness calculations. We calculate total social costs as the sum of: (a) programmatic costs; (b) the cost of public funds; and (c) costs incurred by households in responding to the interventions. From social costs we exclude incentive payments (apart from their contribution the cost of public funds), considering these payments to be transfers (Kremer, Miguel, and Thornton 2009). Incentive payments may also not be considered a cost, but rather simply another way of allocating salary expenditures (Muralidharan and Sundararaman 2011). Note that although we only consider comparative cost-effectiveness in reducing anemia prevalence (the primary outcome of the study), it is possible that the treatments, particularly the block grant, do produce other benefits not considered here. Moreover, if the sole purpose of transfers to schools is to reduce anemia, there may be more cost-effective options than unrestricted block grants. Our goal is not to conduct a full cost-benefit analysis, but rather to compare strategies for reducing anemia. Although we find no intervention effects on standardized exam scores, even these (together with anemia measures) may fail to fully capture intervention benefits.

Table 5 presents these results.^39^ The key finding that we highlight is that although large block grants were as effective in reducing student anemia as large incentives, they were more expensive. First, considering full social costs and using the full sample, the relative cost per case of anemia averted was 1,453 yuan (about $227) in the large incentive/small block grant group—but 44% larger in the large block grant group (2,099 yuan, or about $328).^40^ Second, the cost-effectiveness of these two interventions relative to each other is similar when we restrict our calculations to children anemic at baseline (as we do in Sections 4 and 5, following our preanalysis plan). Specifically, the large incentive/small block grant intervention is approximately 50% more cost effective than large block grant intervention without incentives (723 yuan, or $113, per case of anemia averted vs. 1,447 yuan, or $226). Finally, considering calculating only programmatic costs and using children anemic at baseline, the cost-effectiveness of the large incentive/small block grant intervention is roughly one third of that of the large block grant (114 yuan, or about $18 vs. 331 yuan, or about $52).

**Table 5.** Comparative cost effectiveness calculations.

	Incremental amount relative to comparison (small block grant, no incentives) group					
		Small block grant, small incentives	Small block grant, large incentives	Large block grant, no incentives	Large block grant, small incentives	Large block grant, large incentives
*Panel A: Costs*						
*Programmatic costs*						
(1)	Block grant	0.0	0.0	48.0	48.0	48.0
(2)	Incentive payments	1.1	15.7	0.0	1.4	17.5
*Cost of public funds*						
(3)	Cost of public funds	0.3	4.7	14.4	14.8	19.7
*Costs to households*						
(4)	Full sample	45.5	60.7	90.8	38.3	62.7
(5)	Anemic sample	34.6	95.0	147.5	26.6	49.4
Total costs						
(6)	Programmatic	1.1	15.7	48.0	49.4	65.5
(7)	Social—full sample	45.8	65.4	153.2	101.1	130.3
(8)	Social—anemic sample	34.9	99.7	209.9	89.5	117.0
*Panel B: Anemia reduction (percentage point reduction)*						
(9)	Full sample	0.028	0.045	0.073	0.074	0.032
(10)	Anemic sample	0.012	0.138	0.145	0.199	0.087
*Panel C: Cost effectiveness (cost of averting one anemia case)*						
*Full sample*						
(11)	Programmatic	N.S.	349.6	657.5	668.1	2047.4
(12)	Social	N.S.	1452.7	2098.6	1366.1	4072.4
*Anemic sample*						
(13)	Programmatic	N.S.	114.0	331.0	248.4	753.1
(14)	Social	N.S.	722.5	1447.3	449.6	1345.1

Notes: All costs in renminbi per child (exchange rate as of September 2012 was 6.3 USD/RMB). Costs of the information intervention and anemia testing are excluded as these are constant across treatments. The cost of the information intervention was 1,020 yuan per school and the cost of anemia testing was 6.7 yuan per child. Additional administrative costs are assumed to be negligible as administration of block grants could be built into the administration of other school finances, administrative costs of rewards into administration of existing school administrator evaluation policies and policies, and monitoring of anemia into existing policies stipulating annual checkups for school children. In the absence of good estimates for China (and other developing countries), the cost of public funds is assumed to be 0.3 based on estimates for the United States (Ballard, Shoven, and Whalley, 1985). Social costs include costs incurred by households and exclude incentive payments (except the deadweight loss to taxation) considering them a transfer. Costs to households include costs of purchasing additional food and additional time spent attending parent meetings. Estimates for additional food costs are based on estimates for impact on meat, vegetable, and fruit consumption at home reported in Online Appendix Tables A.5 and A.6. Reported increases in times foods were consumed in the past week are assumed to be constant across all 24 weeks of the program. Serving sizes are assumed to be half of the recommended daily consumption (25 g of meat, 150 g of vegetables, and 100 g of fruit). Food prices are based on prices in local markets as reported by the school accountant at baseline. Time spent in parent meetings is based on estimates in Online Appendix Tables A.7 and A.8. One meeting is assumed to have an opportunity cost of 60 yuan (approximately half of local daily wages). Anemia reduction estimates in panel B are calculated from estimates in Table 2 and Online Appendix Table A.3. Effects not significant (N.S.) for the small block grant, no incentives intervention.

## 6. Conclusion

This paper provides new evidence on how public sector managers respond to the provision of performance incentives. To the best of our knowledge, it is the first study to analyze how behavioral responses to performance pay interact with exogenously assigned levels of resources—a critical issue in the design of incentive systems under stringent resource constraints (as is common in many developing countries).

We report four key findings. First, when school administrators have fewer budgetary resources available to them, large performance incentives (with realized payments equivalent to a couple of months of annual salary) lead to substantial improvement in service delivery. This seems driven by greater effort rather than changes in budgetary resource allocation. In particular, we find evidence that school administrators were able to innovate, working through their students’ parents to alter nutritional practices at home. Second, smaller incentives (one tenth the size of the larger ones) were ineffective on average and had negative effects on prosocially motivated administrators. Third, even absent explicit performance incentives, increasing school administrators’ budgets led to important improvements in performance (but was considerably less cost-effective than using performance incentives), implying the presence of other motives—potentially including intrinsic ones—in our context.

Fourth, we find that performance incentives and unrestricted grants are substitutes in the production of health when incentives are large. The degree of substitution is substantial: at the policy-relevant levels that we study, increasing the size of unrestricted block grants completely crowds out the effect of incentives (and vice versa). This is an important result for resource-poor environments in which both budgetary resources and performance incentives are used simultaneously as policy levers for improving the quality of public service delivery.

There are of course limitations to this study. One is that, as with all empirical studies, our results are not generalizable to settings beyond our study context. However, school-based nutrition programs like the one we study are nearly ubiquitous in low- and middle-income countries (Del Rosso and Marek 1996; Mũkoma and Flisher 2004; Bundy et al. 2006; Orazem et al. 2008). Because a large share of children attend school in most countries, school-based programs are widely considered to be among the most cost-effective means of delivering child health interventions (Orazem et al. 2008). We believe that our study provides behavioral insights relevant in a variety of settings in which managers have budgetary discretion—and increasingly also face high powered incentives. Another is that our study estimates short-run intervention effects. Longer-run effects may differ, particularly as administrators learn more about the relationships among their effort, various inputs, and anemia reduction. Finally, although we find that incentives for school administrators to reduce student anemia were effective, we also note features that may make our setting conducive to the use of performance incentives. One is that the rewarded outcome (anemia reduction) can be measured objectively and reliably. Relative to other settings, frontline workers (teachers in our setting) in China may also be relatively responsive to instruction from administrators, which might alleviate problems of moral hazard in teams that could be more prevalent elsewhere.

Overall, among public sector administrators in rural China, we find evidence that appropriately designed performance incentives (sufficiently large, and absent substantial discretionary resources) can improve public sector service delivery—and ultimately, child outcomes. Despite the bureaucratic environment, our study suggests that performance pay can be an effective approach to motivating public sector managers.

## Supplementary Material

jvy047_Luo_etal_Replication_Files

jvy047_Supplemental_File

## Footnotes

1 Previous studies have shown anemia rates among primary school aged students in poor regions of western China to be around 30% on average (Luo et al. 2011; Miller et al. 2012).

2 One concern in this context may be multitasking–in particular, whether or not incentives to reduce anemia led schools to focus on anemia reduction at the expense of teaching. In a separate analysis of multitasking effects, we find no evidence that these incentives led to a reduction in student test scores on standardized Math and Chinese exams designed by the research team based on the national curriculum and designed to have good psychometric properties. However, we do find some evidence of multitasking on margins of teaching where the costs to administrators and teachers of diverting effort are lower (i.e., tasks that would likely be the first source of diverted effort). For example, in “secondary subjects” (subjects other than math or Chinese, which are not a focus of high school entrance exams and hence given less weight in the curriculum) we find evidence of diverted effort when children were taught these subjects by a homeroom teacher (who was often given responsibilities related to the anemia program).

3 Several recent studies have examined performance pay provided as personal income to front line workers in the health and education sectors, including Lavy (2002, 2009), Glewwe et al. (2010), Muralidharan and Sundararaman (2011), Duflo et al. (2012), Ashraf et al. (2014), and Behrman et al. (2015). Other recent studies have focused on incentives to institutions paid as budget revenue. These include Bloom et al. (2006), Basinga et al. (2011), Gertler and Vermeersch (2012), Olken, Onishi, and Wong (2014), and Yip et al. (2014). Behrman et al. (2015) also study incentives for school administrators, but bundled with incentives to students and teachers.

4 On performance pay for corporate executives and private sector managers in developed countries, see Jensen and Murphy (1990), Hall and Liebman (1998), Murphy (1999), Hall and Murphy (2003), and Oyer and Schaefer (2005). Bandiera et al. (2007) and Bloom et al. (2013) study these virtues of performance pay for private sector managers in developing countries. Burgess et al. (2010) study a team-based incentive scheme in the context of a government agency in the United Kingdom; although the scheme was not explicitly targeted to managers, the authors find that the team-based incentive induced managers to reallocate more efficient workers to incentivized tasks.

5 The two exceptions of which we are aware are Singh (2015) and Miller et al. (2012). Though not studying the health impacts of incentives tied to health outcomes, Leonard (2003) studies traditional healers’ use of outcome-contingent contracts in Cameroun. In the education sector, performance pay rewarding good test scores is more common (Lavy 2009; Muralidharan and Sundararaman 2011; Behrman et al. 2015).

6 Kremer and Miguel (2007), Thornton (2008), Banerjee et al. (2010), Cohen and Dupas (2010), Karlan, List, and Shafir (2011), Duflo, Kremer, and Robinson (2011).

7 See Gneezy, Meier, and Rey-Biel (2011) for several examples.

8 We also note at least two key differences with this previous literature: one is that receiving incentive payments in our study required sustained behavior change (reducing iron deficiency anemia requires several months of dietary chance), and another is that because all school administrators receive information about anemia, the presence of incentives may not alter its salience as much (Benhassine et al. 2015).

9 See Rogger (2014) and Nath (2015) for other models of bureaucratic behavior with choices of effort.

10 Note that the third order derivatives of the production function include *f_eee_, f_AAA_, f_ee__A_, and f_e__AA_*; the solution would be very cumbersome if we did not assume that these are zero.

11 Note that if *S*΄΄΄< 0, the term *S*΄΄΄*f_eA_*, which is part of *de*/*dtdG* (see the Online Appendix), can partially offset this effect.

12 Note that if one assumed a simple power function such as *S*(*G* − *A*) = (*G* − *A*)^β^, the condition that *S*^΄΄^ < 0, would also imply necessarily that *S*΄΄΄> 0. However, this would not be the case for other functional forms, such as a cubic polynomial.

13 There is some debate in the public health literature on the proportion of the anemia burden attributable to iron deficiency (Balarajan et al. 2011). Intestinal worms are unlikely to be a major cause of anemia in our study areas as the prevalence of hookworm (the parasite most commonly associated with anemia) is low (Xu et al. 1995).

14 Previous trials addressing iron deficiency and anemia have suffered from low levels of compliance or attempted to preempt compliance problems. Bobonis et al. (2006), for example, instructed preschool teachers to provide children with iron therapy for 30 days following health camps but found that only around 18 days were actually administered. The WISE study in Indonesia (Thomas et al. 2006) hired facilitators to regularly visit participants and remind them to take their supplements.

15 A lower bound of 150 students was chosen to ensure that the number of samples students per school was enough to meet power requirements. 300 was chosen as the upper bound to keep the project within budget. These bounds are on reported school sizes; actual numbers of students are often significantly less than reported. Note that 39.9% of rural primary schools in the sampling frame (all rural primary schools in project counties) were reported to be within this range.

16 Local administration of schools is generally done at the school district level, which is below the township. Contamination due to two school administrators meeting at events organized at higher levels, for example, was thus unlikely.

17 Using data from Miller et al. (2012), we performed Monte Carlo simulations to conduct power calculations for students who were anemic at the time of that study's baseline survey. The intraclass correlation was estimated adjusting for covariates (baseline hemoglobin concentration, the number of students in each school, whether schools had a kitchen, student–teacher ratio, distance to the furthest village served by the school, percent boarding students, and county dummies), which we also specified as covariates in the current study's preanalysis plan.

18 Information on food consumption was collected using a seven-day recall “food frequency questionnaires” (FFQs) completed by students as part of the endline survey. These questionnaires asked children the number of times they had eaten each of 33 food items in the past seven days, separately for school and home. Food frequency questionnaires (FFQs) have long been used in nutrition research and have been recommended for use in large surveys of children given low cost and low respondent burden (McPherson et al. 2000; Magarey et al. 2011). FFQ responses by children about their own consumption has been shown to be more accurate than the responses of their parents (Burrows et al. 2013).

19 For budgetary reasons, household surveys were given to students to take home and return. As a result, household forms are missing for approximately 20% of students. All possible information on students and households was collected with the student survey, which was administered by enumerators.

20 Thus, all schools in the experiment received a small or large grant. This was done to ensure that all schools had access to resources that could be devoted to anemia reduction. In a previous study, we find that educating school administrators on anemia (including the same information as in the current study) alone, without incentives or grants, had no detectable impact on anemia rates (Miller et al. 2012). Our reference group also mimics how a recent Chinese school nutrition program (costing 16 billion yuan per year) was designed. Under the program, local education bureaus and schools receive 3 yuan per day per student (4 yuan for boarding students) to provide nutritious meals. How exactly the program is implemented and monitored varies widely across localities.

21 We measured anemia using a sample of 50 fourth and fifth graders and calculated the implied number of anemic children in the school using the prevalence rate in our sample. Although administrators could possibly discern which students were tested at baseline (although testing was done before contract assignment), they were later told explicitly that another sample of students would be drawn at the end of the school year. Moreover, unreported analyses show that teachers were unable to correctly name or identify anemic students at endline.

22 We use a conversion rate of $1 = 6.4 RMB, the approximate exchange rate at the time of the baseline survey (September 2011).

23 There are presumably superior contract structures, but optimal contract design requires substantial information not available to us, including information about the cost of provider effort, the productivity of provider effort, and the utility functions of both providers and the contracting “principal” (Laffont and Tirole 1993; Salanié 2005). Simple, easily understandable contracts may also appear more transparent to school administrators and promote credibility.

24 We did reveal the identity of students who were severely anemic (with hemoglobin concentration below 80 g/L) as these students required immediate medical attention. There were 3 such students found at baseline.

25 Note that administrators in all study cells were provided the same information about the number of anemic children in their respective schools.

26 After explaining block grant assignment to administrators, we asked them to complete a nonbinding budget plan for how they intended to use the block grant. Our study team emphasized that this plan was nonbinding, but this plan would be used to coordinate orders for iron supplements to be delivered to schools. This was necessary because the market for supplements in rural areas is limited. Administrators were free to change their supplement orders at any time. Administrators had no reason to believe that second installments were conditional on performance—they were given an explicit time frame and told an explicit amount for the second installment.

27 Note that although these transfers were not large compared to total school expenditures, they do represent a significant increase in budgetary autonomy for school administrators as the bulk of school expenditures are earmarked for specified uses at higher levels of administration. Although administrators often have discretion over small expenditures, larger expenses require approval from upper levels.

28 This table shows summary statistics and tests balance for our main analysis sample of students initially anemic at baseline. Summary statistics and balance tests for the full sample are given in Online Appendix Table A.1.

29 Although 170 schools were included in the study, no students were found to be anemic in 3 schools at baseline. No schools refused participation in the study. The baseline anemia rate (defined as Hb < 120 g/L) in the full sample was 24%. Online Appendix Table A.11 shows transitions in and out of anemia status in the comparison (small grant only) group.

30 These tests were conducted by regressing treatment status on all 15 baseline covariates and testing that the coefficients were jointly zero. The smallest *p*-value from these *F*-tests was 0.29.

31 This analysis plan was filed with the Abdul Latif Jameel Poverty Action Lab at http://www.povertyactionlab.org/Hypothesis-Registry.

32 We did not prespecify the last of these (whether or not the joint effect of the large grant and large incentive is negative), but we did prespecify whether or not incentives and large grants are complements or substitutes (β_4_ = 0,   β_5_ = 0).

33 Table 3 reports results for students found to be anemic at baseline as pre-specified. Appendix Table 3 shows main results for the full sample.

34 Likewise, β_3_ + β_5_, the marginal effect of increasing the grant amount given large incentives is insignificant (β_3_ + β_5_ = 0.051, adjusted *p*-value 0.742).

35 Subindices for supplements and food (including separate indices for food at home and school) were not explicitly specified in the preanalysis plan. Online Appendix Table A.9 reproduces Table 4, including additional tests of linear combinations of coefficients estimated using equation (19). These additional tests were not prespecified.

36 Specifically, the number of individual meetings between administrators and households over the past semester increased by 0.52 (column (2), row 2)—an increase of 59%; whether or not schools contacted parents about nutrition in the past semester rose by 12 percentage points (column (3), row 2)—an increase of 29%; and whether or not schools contacted parents about feeding children iron-rich foods rich in the past semester rose by 10 percentage points (column (4), row 2)—an increase of 47%. Note that the number of school-wide parent meetings and number of individual meetings with parents were not prespecified to be part of this index.

37 We speculate that the bureaucratic environment is one reason that administrators chose to work through households. Administrators may have viewed this strategy as a way to reduce anemia (and increase rewards) while avoiding the risk of career harm due to possible adverse events. This career harm may also be more severe under incentives if incentives altered perceptions of administrators’ motivation for reducing anemia (analogous to how incentives may crowd-out effort if they alter the motives for prosocial tasks perceived by others—Bénabou and Tirole (2006)).

38 Given our experimental design, we are unable to rule out the possibility of complementarity at lower levels of resources.

39 See table notes for further details about these calculations.

40 These estimates exclude administrative costs of the incentive scheme (assuming these would be rolled into existing policies as noted in the notes to Table 5). When the costs incurred for anemia testing (6.7 RMB per child in a sample of 50 children per school annually) are included in costs of the incentive scheme, the large incentive/small block grant remains more socially cost-effective than the large block grant at 1,848 yuan per case averted.

## Notes

*The editor in charge of this paper was M. Daniele Paserman*.
