# Supplementary material for: Can Bureaucrats Really Be Paid Like Ceos? Substitution Between Incentives and Resources Among School Administrators in China
Source: J Eur Econ Assoc. 2019 Jan 17;18(1):165–201. doi: 10.1093/jeea/jvy047 (PMC7053554; doi:10.1093/jeea/jvy047)
Supplement: jvy047_Luo_etal_Replication_Files [file jvy047_luo_etal_replication_files.zip › replication files/readme.pdf]

Replication files for “CAN BUREAUCRATS REALLY BE PAID LIKE CEOS? SUBSTITUTION BETWEEN INCENTIVES AND RESOURCES AMONG SCHOOL ADMINISTRATORS IN CHINA”

Datasets:
data.dta: Student-level dataset containing all variables used in the analysis apart from school-level budget data.

budget.dta: School-level budget dataset for figure 4.

Programs:
Bureaucrats.do: Reproduces all figures and tables in the paper and online appendix.

fsdrm.ado: Program to compute adjusted p-values controlling the Family Wise Error Rate using the ste-down method of Westfall and Young (1993).
