# Supplementary material for: Can Bureaucrats Really Be Paid Like Ceos? Substitution Between Incentives and Resources Among School Administrators in China
Source: J Eur Econ Assoc. 2019 Jan 17;18(1):165–201. doi: 10.1093/jeea/jvy047 (PMC7053554; doi:10.1093/jeea/jvy047)
Supplement: jvy047_Supplemental_File [file jvy047_supplemental_file.pdf]

ONLINE APPENDIX TO "CAN BUREAUCRATS REALLY BE PAID LIKE CEOS? SUBSTITUTION BETWEEN INCENTIVES AND RESOURCES AMONG SCHOOL ADMINISTRATORS IN CHINA"

Renfu Luo
Peking University

Grant Miller
Stanford University

Scott Rozelle
Stanford University

Sean Sylvia
University of North Carolina at Chapel Hill

Marcos Vera-Hernández
University College London

E-mail: luorf@pku.edu.cn (Luo); ngmiller@stanford.edu (Miller); rozelle@stanford.edu (Rozelle); sean_sylvia@unc.edu (Sylvia); m.vera@ucl.ac.uk (Vera-Hernández)

Journal of the European Economic Association
Preprint prepared on 17 October 2018 using jeea.cls *v1.0.*

Appendix A: Appendix Tables

Table A.1: Descriptive Statistics and Balance Check (Full sample)

	No Incentive, Small Grant Group		Coefficient (standard error) on:						
	Mean	SD	Small Incentive	Large Incentive	Large Grant	(Small-Incentive)X (Large Grant)	(Large Incentive)X (Large Grant)	Joint Test P-value: All Coefficients=0	Observations
	(1)	(2)	(3)	(4)	(5)	(6)	(7)	(8)	(9)
Panel A: Child Characteristics									
(1) Hemoglobin Concentration (g/L)	134.191	12.912	-0.912<br>(1.127)	-1.192<br>(1.009)	0.514<br>(1.028)	0.140<br>(1.501)	-0.021<br>(1.476)	0.541	8398
(2) Anemic (0/1)	0.233	0.423	0.024<br>(0.017)	0.017<br>(0.019)	-0.015<br>(0.018)	-0.001<br>(0.024)	0.003<br>(0.025)	0.222	8398
(3) Age (years)	10.713	1.173	-0.172<br>(0.128)	-0.041<br>(0.111)	-0.030<br>(0.106)	0.352*<br>(0.185)	-0.013<br>(0.144)	0.379	8398
(4) 5th Grade (0/1)	0.531	0.499	-0.002<br>(0.006)	0.001<br>(0.006)	-0.005<br>(0.008)	0.007<br>(0.011)	0.001<br>(0.010)	0.941	8398
(5) Female (0/1)	0.485	0.500	0.003<br>(0.020)	-0.008<br>(0.017)	-0.009<br>(0.019)	0.024<br>(0.030)	0.010<br>(0.025)	0.808	8398
(6) Times Consumed Meat in Past Week (incl. Chicken, Pork, Beef, Lamb)	3.826	3.966	-0.194<br>(0.435)	-1.174***<br>(0.316)	-0.402<br>(0.442)	-0.432<br>(0.604)	0.962*<br>(0.550)	0.002***	8398
Panel B: School Characteristics									
(7) Number of Students	207.094	64.823	-1.276<br>(17.567)	3.623<br>(14.959)	-5.396<br>(16.043)	25.344<br>(25.554)	12.357<br>(20.856)	0.797	170
(8) Has Kitchen (0/1)	0.063	0.246	0.141<br>(0.101)	0.074<br>(0.075)	0.059<br>(0.083)	-0.075<br>(0.162)	-0.068<br>(0.120)	0.681	170
(9) Student-Teacher Ratio	16.228	4.227	2.538*<br>(1.354)	0.893<br>(1.210)	-0.286<br>(1.159)	-1.506<br>(1.911)	1.064<br>(1.657)	0.257	170
(10) Time to Furthest Village Served (mins)	62.031	36.695	12.218<br>(13.109)	-2.281<br>(11.564)	3.878<br>(12.945)	-7.346<br>(21.467)	3.764<br>(17.794)	0.921	170
(11) Percent Boarding Students (%)	5.327	11.404	1.511<br>(4.112)	0.106<br>(3.006)	0.610<br>(3.492)	-0.079<br>(6.293)	-1.611<br>(5.179)	0.991	170
Panel C. School Administrator Characteristics									
(12) Male (0/1)	0.938	0.246	0.015<br>(0.058)	0.056<br>(0.041)	0.065<br>(0.042)	-0.012<br>(0.059)	-0.093*<br>(0.051)	0.488	170
(13) Age (years)	39.313	7.253	1.883<br>(2.047)	1.620<br>(1.777)	1.892<br>(1.831)	-5.022*<br>(2.957)	-0.399<br>(2.560)	0.351	170
(14) Higher Education Degree (0/1)	0.906	0.296	0.002<br>(0.089)	-0.022<br>(0.078)	-0.122<br>(0.090)	0.047<br>(0.133)	0.010<br>(0.122)	0.506	170
(15) Experience (years)	8.031	6.141	-0.242<br>(1.472)	1.088<br>(1.729)	0.838<br>(1.569)	-2.706<br>(2.156)	-0.310<br>(2.520)	0.141	170
(16) Monthly Base Salary (yuan)	1854.750	692.449	-48.275<br>(190.039)	-103.680<br>(175.240)	-26.684<br>(178.777)	-321.983<br>(304.280)	-17.503<br>(242.283)	0.641	170

NOTES. Data source: baseline survey. Table uses full sample of children tested for hemoglobin concentration. Children are considered anemic if they have an altitude-adjusted hemoglobin concentration below 120 g/L (per WHO guidelines). The first and second columns show the mean and standard deviation in the comparison (small grant, no incentives) group. Columns 3 through 7 show coefficients and standard errors from a regression of each characteristic on indicators for incentive and large grant treatment group indicators and there interactions, controlling for randomization strata. Column 8 shows p-values from a test that coefficients are jointly zero. *, **, and *** indicate significance at 10%, 5% and 1%.

Table A.2: Attrition

Dependent Variable:		Hb Measurement Missing at Endline		Household Survey Missing at Endline	
		(1)	(2)	(3)	(4)
Panel A: Treatments and Interactions					
(1)	B1: Small Incentive	0.014	0.017	0.164	0.176
		(0.019)	(0.020)	(0.100)	(0.099)
		[0.473]	[0.404]	[0.102]	[0.078]
		{0.892}	{0.851}	{0.546}	{0.529}
(2)	B2: Large Incentive	-0.027	-0.027	0.124	0.133
		(0.017)	(0.017)	(0.081)	(0.081)
		[0.127]	[0.117]	[0.126]	[0.102]
		{0.545}	{0.546}	{0.546}	{0.529}
(3)	B3: Large Grant	0.010	0.009	0.019	0.042
		(0.023)	(0.022)	(0.098)	(0.104)
		[0.647]	[0.703]	[0.846]	[0.689]
		{0.893}	{0.932}	{0.890}	{0.931}
(4)	B4: (Small Incentive)X(Large Grant)	-0.059	-0.063	-0.233	-0.265
		(0.031)	(0.031)	(0.139)	(0.149)
		[0.056]	[0.042]	[0.095]	[0.077]
		{0.400}	{0.372}	{0.546}	{0.529}
(5)	B5: (Large Incentive)X(Large Grant)	-0.007	0.000	0.069	0.044
		(0.031)	(0.031)	(0.140)	(0.139)
		[0.835]	[0.992]	[0.626]	[0.750]
		{0.893}	{0.994}	{0.890}	{0.931}
Panel B: Child Characteristics					
(6)	Baseline Hemoglobin Concentration (g/L)	-0.000	-0.000	0.001	0.001
		(0.001)	(0.001)	(0.001)	(0.001)
(7)	Age (years)		0.013*		-0.010
			(0.007)		(0.009)
(8)	5th Grade (0/1)		-0.011		0.007
			(0.011)		(0.023)
(9)	Female (0/1)		-0.018		-0.019
			(0.013)		(0.014)
Panel C: School Characteristics					
(10)	Number of Students		-0.000		0.001
			(0.000)		(0.001)
(11)	Has Kitchen (0/1)		0.006		0.143
			(0.025)		(0.112)
(12)	Student-Teacher Ratio		-0.001		-0.006
			(0.002)		(0.007)
(13)	Time to Furthest Village Served (mins)		-0.000		0.000
			(0.000)		(0.001)
(14)	Percent Boarding Students (%)		-0.000		-0.003
			(0.001)		(0.003)
(15)	"Free Lunch" Policy School		0.019		-0.167
			(0.056)		(0.258)
(16)	Constant	0.118	0.027	-0.095	0.117
		(0.103)	(0.124)	(0.243)	(0.412)
(17)	Observations	2051	2051	1923	1923
(18)	R-squared	0.046	0.051	0.334	0.347
(19)	Mean in No Incentive, Small Grant Group	0.087		0.154	

NOTES. Table uses sample of children testing anemic at baseline. Children are considered anemic if they have an altitude-adjusted hemoglobin concentration below 120 g/L (per WHO guidelines). The dependent variable in columns 1 and 2 is a dummy variable indicating missing hemoglobin measurements at endline. The dependent variable in columns 3 and 4 is a dummy variable indicating missing household forms at endline conditional on a child's hemoglobin measurement being non-missing. In addition to what is shown regressions also control for county and randomization strata fixed effects. Standard errors are shown in parentheses, unadjusted p-values are shown in square brackets and p-values adjusted for multiple inference are shown in curly brackets. Adjusted p-values were constructed using the free step-down resampling method of Westfall and Young (1993) with 10,000 iterations. *, **, and *** indicate significance at 10%, 5% and 1%. based on adjusted p-values.

Table A.3: Effects of School Administrator Anemia Reduction Incentives and Block Grant Size on Student Hemoglobin Concentration and Anemia Prevalence (Full Sample)

Dependent Variable:		Anemic at Endline (Hb<120 g/L)	Hemoglobin Concentration (g/L)
		(1)	(2)
Panel A: Treatment Effect Regressions			
(1)	B1: Small Incentive	-0.028	1.054
		(0.020)	(0.987)
		[0.163]	[0.287]
		{0.587}	{0.747}
(2)	B2: Large Incentive	-0.045	0.918
		(0.022)	(0.946)
		[0.046]	[0.333]
		{0.373}	{0.767}
(3)	B3: Large Grant	-0.073**	2.872
		(0.021)	(0.989)
		[0.001]	[0.004]
		{0.049}	{0.117}
(4)	B4: (Small Incentive)X(Large Grant)	0.027	-0.857
		(0.027)	(1.340)
		[0.321]	[0.523]
		{0.647}	{0.829}
(5)	B5: (Large Incentive)X(Large Grant)	0.086	-3.312
		(0.031)	(1.404)
		[0.006]	[0.019]
		{0.149}	{0.235}
(6)	Observations	7945	7945
(7)	Mean in No Incentive, Small Grant Group	0.176	136.334
Panel B: P-values for Additional Hypotheses			
(8)	B1–B2: Effect of Large Incentive vs. Effect of Small Incentive given Small Grant	[0.383]	[0.884]
		{0.647}	{0.908}
(9)	B2–B3: Effect of Large Incentive given Small Grant vs. Effect of Increasing Grant Amount	[0.146]	[0.036]
		{0.587}	{0.301}
(10)	B2 + B5: Effect of Large Incentive given Large Grant	[0.038]	[0.013]
		{0.373}	{0.209}

NOTES. Table uses full sample of children tested for hemoglobin concentration. Rows 1-5 in Panel A show estimated coefficients for treatment group indicators and interactions obtained by estimating equation (12) (controlling for baseline hemoglobin concentration, student age, student grade, student sex, number of students in the school, whether the school has a canteen, student teacher ratio, distance to the furthest village served, percent of boarding students, whether the school has implemented the "Free Lunch" policy, county dummy variables, and dummy variables for randomization strata). Standard errors are shown in parentheses, unadjusted p-values are shown in square brackets and p-values adjusted for multiple inference are shown in curly brackets. Adjusted p-values were constructed using the free step-down resampling method of Westfall and Young (1993) with 10,000 iterations. *, **, and *** indicate significance at 10%, 5% and 1%. based on adjusted p-values. Panel B shows unadjusted and adjusted p-values from tests between coefficients.

Table A.4: Child and Household Reported Receipt of Supplements and Iron-Rich Food (Full Sample)

Dependent Variable:	Index: Supplements and Food	Sub-index: Supplements	Sub-index: Food	Sub-index: Food at School	Sub-index: Food at Home	Index: Information	Sub-index: Information to Students	Sub-index: Information to Households
	(1)	(2)	(3)	(4)	(5)	(6)	(7)	(8)
(1) B1: Small Incentive	0.109*	0.191*	0.013	-0.039	0.126	0.079	0.197	-0.002
	(0.045)	(0.072)	(0.037)	(0.049)	(0.049)	(0.069)	(0.116)	(0.076)
	[0.015]	[0.008]	[0.722]	[0.429]	[0.011]	[0.250]	[0.092]	[0.984]
	{0.062}	{0.083}	{0.771}	{0.854}	{0.126}	{0.504}	{0.390}	{0.986}
(2) B2: Large Incentive	0.118*	0.191*	0.045	0.021	0.130	0.116	0.199	0.131
	(0.044)	(0.075)	(0.039)	(0.053)	(0.047)	(0.065)	(0.086)	(0.084)
	[0.008]	[0.011]	[0.249]	[0.695]	[0.007]	[0.073]	[0.022]	[0.120]
	{0.062}	{0.083}	{0.665}	{0.854}	{0.107}	{0.394}	{0.204}	{0.519}
(3) B3: Large Grant	0.173***	0.247***	0.094	0.107	0.100	0.197*	0.226	0.187
	(0.041)	(0.060)	(0.047)	(0.064)	(0.053)	(0.067)	(0.095)	(0.097)
	[<0.001]	[<0.001]	[0.049]	[0.097]	[0.062]	[0.004]	[0.019]	[0.055]
	{0.005}	{0.008}	{0.346}	{0.503}	{0.293}	{0.079}	{0.204}	{0.410}
(4) B4: (Small Incentive)X(Large Grant)	-0.226**	-0.364**	-0.066	-0.056	-0.101	-0.123	-0.037	-0.216
	(0.061)	(0.100)	(0.057)	(0.077)	(0.076)	(0.113)	(0.165)	(0.132)
	[<0.001]	[<0.001]	[0.249]	[0.464]	[0.187]	[0.280]	[0.822]	[0.105]
	{0.013}	{0.016}	{0.665}	{0.854}	{0.443}	{0.504}	{0.859}	{0.519}
(5) B5: (Large Incentive)X(Large Grant)	-0.179*	-0.259*	-0.101	-0.13	-0.106	-0.139	-0.158	-0.200
	(0.062)	(0.095)	(0.067)	(0.086)	(0.077)	(0.094)	(0.129)	(0.142)
	[0.004]	[0.007]	[0.129]	[0.130]	[0.169]	[0.144]	[0.221]	[0.161]
	{0.053}	{0.083}	{0.531}	{0.535}	{0.443}	{0.490}	{0.526}	{0.519}
(6) Observations	7965	7959	7949	7894	7947	7961	7943	6484
(7) Mean in No Incentive, Small Grant Group	0.0137	-0.118	0.043	0.034	0.008	-0.108	-0.152	-0.084

NOTES. Table uses full sample of children tested for hemoglobin concentration. Rows 1-5 show estimated coefficients for treatment group indicators and interactions obtained by estimating equation (12) (controlling for the baseline value of the dependent variable, student age, student grade, student sex, number of students in the school, whether the school has a canteen, student teacher ratio, distance to the furthest village served, percent of boarding students, whether the school has implemented the "Free Lunch" policy, county dummy variables, and dummy variables for randomization strata). The dependent variable in each regression is a summary index constructed using the GLS weighting procedure in Anderson (2008). Estimates for the individual components of each index are shown in Appendix Tables 5 and 6. Standard errors are shown in parentheses, unadjusted p-values are shown in square brackets and p-values adjusted for multiple inference are shown in curly brackets. Adjusted p-values were constructed using the free step-down resampling method of Westfall and Young (1993) with 10,000 iterations. *, **, and *** indicate significance at 10%, 5% and 1% based on adjusted p-values.

Table A.5: Child and Household Reported Receipt of Supplements and Iron-Rich Food, Index Components

Index:	Supplements						Food					
Sub-index:							Food at School			Food at Home		
Dependent Variable:	Household received supplements to give to child (Household Response)	School provided supplements to children (Child Response)	Times per week supplements distributed by school (Child Response)	School provided supplements to take home over the weekend (Child Response)	All classmates take supplements (Child Response)	Days given supplements last month (Child Response)	Times consumed meat at school in past week	Times consumed green vegetables at school in past week	Times consumed fruit in school in past week	Times consumed meat at home in past week	Times consumed green vegetables at home in past week	Times consumed fruit at home in past week
	(1)	(2)	(3)	(4)	(5)	(6)	(7)	(8)	(9)	(10)	(11)	(12)
(1) B1: Small Incentive	0.042	0.200	0.805	-0.025	-0.314	0.030	0.044	-0.564	-0.18	0.413	0.616	0.455
	(0.101)	(0.065)	(0.439)	(0.075)	(0.522)	(1.774)	(0.197)	(0.3)	(0.2)	(0.403)	(0.711)	(0.562)
	[0.679]	[0.002]	[0.068]	[0.738]	[0.549]	[0.987]	[0.825]	[0.062]	[0.371]	[0.307]	[0.387]	[0.419]
	{1.000}	{0.569}	{0.995}	{1.000}	{1.000}	{1.000}	{0.949}	{0.43}	{0.73}	{1.000}	{1.000}	{1.000}
(2) B2: Large Incentive	0.262	0.179	0.109	-0.084	-0.490	-0.350	0.201	0.153	-0.005	1.120	1.393	0.988
	(0.086)	(0.061)	(0.444)	(0.057)	(0.426)	(1.759)	(0.186)	(0.242)	(0.169)	(0.364)	(0.705)	(0.563)
	[0.003]	[0.004]	[0.806]	[0.147]	[0.252]	[0.843]	[0.28]	[0.528]	[0.975]	[0.002]	[0.050]	[0.081]
	{0.596}	{0.661}	{1.000}	{1.000}	{1.000}	{1.000}	{0.811}	{0.858}	{0.982}	{0.580}	{0.988}	{0.996}
(3) B3: Large Grant	0.108	0.192	0.300	0.045	-0.675	2.732	0.105	0.502	0.488	1.048	1.634	1.039
	(0.093)	(0.075)	(0.435)	(0.066)	(0.435)	(1.877)	(0.199)	(0.298)	(0.211)	(0.393)	(0.831)	(0.651)
	[0.250]	[0.012]	[0.491]	[0.499]	[0.123]	[0.147]	[0.598]	[0.094]	[0.022]	[0.008]	[0.051]	[0.113]
	{1.000}	{0.866}	{1.000}	{1.000}	{1.000}	{1.000}	{0.949}	{0.43}	{0.222}	{0.801}	{0.988}	{0.999}
(4) B4: (Small Incentive)X(Large Grant)	-0.073	-0.445*	-1.310	-0.042	0.265	1.084	0.097	0.05	-0.794	-0.975	-1.160	-0.853
	(0.142)	(0.107)	(0.681)	(0.081)	(0.727)	(2.751)	(0.289)	(0.442)	(0.309)	(0.569)	(1.118)	(0.970)
	[0.607]	[0.000]	[0.056]	[0.599]	[0.716]	[0.694]	[0.738]	[0.911]	[0.011]	[0.088]	[0.301]	[0.381]
	{1.000}	{0.094}	{0.991}	{1.000}	{1.000}	{1.000}	{0.949}	{0.933}	{0.173}	{0.997}	{1.000}	{1.000}
(5) B5: (Large Incentive)X(Large Grant)	-0.388	-0.332	-0.398	0.025	0.383	0.117	-0.455	-0.723	-0.485	-1.620	-1.721	-2.146
	(0.140)	(0.093)	(0.621)	(0.080)	(0.609)	(2.517)	(0.253)	(0.392)	(0.293)	(0.598)	(1.136)	(0.892)
	[0.006]	[0.000]	[0.522]	[0.753]	[0.530]	[0.963]	[0.074]	[0.067]	[0.1]	[0.007]	[0.132]	[0.017]
	{0.754}	{0.283}	{1.000}	{1.000}	{1.000}	{1.000}	{0.468}	{0.43}	{0.46}	{0.781}	{0.999}	{0.910}
(6) Observations	1496	1909	1920	1910	1842	1920	1884	1889	1883	1924	1924	1925
(7) Mean in No Incentive, Small Grant Group	0.500	0.842	3.216	0.152	1.506	8.915	0.674	1.137	1.089	3.837	11.519	7.414

NOTES. Table uses sample of children testing anemic at baseline. Children are considered anemic if they have an altitude-adjusted hemoglobin concentration below 120 g/L (per WHO guidelines). Rows 1-5 show estimated coefficients for treatment group indicators and interactions obtained by estimating equation (12) (controlling for the baseline value of the dependent variable, student age, student grade, student sex, number of students in the school, whether the school has a canteen, student teacher ratio, distance to the furthest village served, percent of boarding students, whether the school has implemented the "Free Lunch" policy, county dummy variables, and dummy variables for randomization strata). Standard errors are shown in parentheses, unadjusted p-values are shown in square brackets and p-values adjusted for multiple inference across all tests corresponding to each index are shown in curly brackets. Adjusted p-values were constructed using the free step-down resampling method of Westfall and Young (1993) with 10,000 iterations. *, **, and *** indicate significance at 10%, 5% and 1% based on adjusted p-values.

Table A.6: Child and Household Reported Receipt of Supplements and Iron-Rich Food, Index Components, Full Sample

Index:	Supplements						Food					
Sub-index:							Food at School			Food at Home		
Dependent Variable:	Household received supplements to give to child (Household Response)	School provided supplements to children (Child Response)	Times per week supplements distributed by school (Child Response)	School provided supplements to take home over the weekend (Child Response)	All classmates take supplements (Child Response)	Days given supplements last month (Child Response)	Times consumed meat at school in past week	Times consumed green vegetables at school in past week	Times consumed fruit in school in past week	Times consumed meat at home in past week	Times consumed green vegetables at home in past week	Times consumed fruit at home in past week
	(1)	(2)	(3)	(4)	(5)	(6)	(7)	(8)	(9)	(10	(11)	(12)
(1) B1: Small Incentive	0.049	0.168	0.751	0.099	-0.105	2.194	0.055	-0.229	-0.144	0.686	0.824	0.915
	(0.092)	(0.061)	(0.441)	(0.057)	(0.424)	(1.555)	(0.204)	(0.302)	(0.198)	(0.309)	(0.492)	(0.398)
	[0.598]	[0.006]	[0.090]	[0.084]	[0.804]	[0.160]	[0.787]	[0.45]	[0.469]	[0.028]	[0.096]	[0.023]
	{1.000}	{0.646}	{0.993}	{0.993}	{1.000}	{0.999}	{0.985}	{0.962}	{0.681}	{0.912}	{0.993}	{0.887}
(2) B2: Large Incentive	0.215	0.128	0.427	-0.022	-0.480	1.596	0.328	-0.019	-0.165	0.732	0.708	0.799
	(0.066)	(0.066)	(0.477)	(0.047)	(0.304)	(1.791)	(0.211)	(0.239)	(0.175)	(0.271)	(0.464)	(0.398)
	[0.001]	[0.052]	[0.372]	[0.634]	[0.116]	[0.374]	[0.123]	[0.937]	[0.346]	[0.008]	[0.129]	[0.046]
	{0.344}	{0.976}	{1.000}	{1.000}	{0.998}	{1.000}	{0.558}	{0.999}	{0.681}	{0.699}	{0.997}	{0.965}
(3) B3: Large Grant	0.161	0.153	0.567	0.071	-0.273	4.394	0.011	0.222	0.502	0.565	0.062	1.053
	(0.077)	(0.065)	(0.418)	(0.048)	(0.336)	(1.758)	(0.248)	(0.342)	(0.233)	(0.310)	(0.521)	(0.438)
	[0.038]	[0.019]	[0.177]	[0.136]	[0.417]	[0.013]	[0.965]	[0.516]	[0.032]	[0.070]	[0.905]	[0.017]
	{0.955}	{0.866}	{1.000}	{0.998}	{1.000}	{0.822}	{0.985}	{0.962}	{0.27}	{0.987}	{1.000}	{0.851}
(4) B4: (Small Incentive)X(Large Grant)	-0.168	-0.402*	-1.452	-0.192	-0.021	-2.005	0.103	0.066	-0.482	-0.633	-0.179	-0.991
	(0.121)	(0.098)	(0.602)	(0.069)	(0.532)	(2.498)	(0.315)	(0.426)	(0.331)	(0.452)	(0.745)	(0.637)
	[0.166]	[0.000]	[0.017]	[0.006]	[0.969]	[0.423]	[0.743]	[0.878]	[0.147]	[0.163]	[0.810]	[0.122]
	{0.999}	{0.063}	{0.857}	{0.646}	{1.000}	{1.000}	{0.985}	{0.999}	{0.538}	{0.999}	{1.000}	{0.997}
(5) B5: (Large Incentive)X(Large Grant)	-0.272	-0.263	-0.641	-0.033	0.032	-2.117	-0.382	0.026	-0.435	-0.637	0.529	-1.359
	(0.110)	(0.088)	(0.626)	(0.067)	(0.421)	(2.600)	(0.321)	(0.408)	(0.282)	(0.456)	(0.743)	(0.578)
	[0.014]	[0.003]	[0.307]	[0.629]	[0.939]	[0.417]	[0.235]	[0.95]	[0.124]	[0.164]	[0.478]	[0.020]
	{0.833}	{0.499}	{1.000}	{1.000}	{1.000}	{1.000}	{0.709}	{0.999}	{0.538}	{0.999}	{1.000}	{0.869}
(6) Observations	6271	7853	7902	7829	7650	7857	7740	7776	7772	7944	7944	7938
(7) Mean in No Incentive, Small Grant Group	0.429	0.799	2.900	0.132	1.245	6.839	0.879	0.937	1.029	4.118	12.336	7.610

NOTES. Table uses full sample of children tested for hemoglobin concentration. Rows 1-5 show estimated coefficients for treatment group indicators and interactions obtained by estimating equation (12) (controlling for the baseline value of the dependent variable, student age, student grade, student sex, number of students in the school, whether the school has a canteen, student teacher ratio, distance to the furthest village served, percent of boarding students, whether the school has implemented the "Free Lunch" policy, county dummy variables, and dummy variables for randomization strata). Standard errors are shown in parentheses, unadjusted p-values are shown in square brackets and p-values adjusted for multiple inference across all tests corresponding to each index are shown in curly brackets. Adjusted p-values were constructed using the free step-down resampling method of Westfall and Young (1993) with 10,000 iterations. *, **, and *** indicate significance at 10%, 5% and 1% based on adjusted p-values.

Table A.7: Nutritional Information to Students and Households, Index Components

Index:	Information to Households						Information to Students				
Dependent Variable:	Number of school-wide parent meetings attended this semester (Household response)	Number of individual meetings with teacher or administrator this semester (Household response)	School contacted household about student nutrition this semester (Household response)	Household told to give student foods rich in iron (Household response)	Parent reports knowing of anemia (Household response)	Parent correctly identifies foods that can prevent anemia (iron-rich foods) (Household response)	School provided nutritional information to student (Student reported)	Times school spoke with students about nutrition in past semester (Student reported)	Students told to eat meat (Student reported)	Student reports knowing of anemia (Student reported)	School provided information to students on anemia (Student reported)
	(1)	(2)	(3)	(4)	(5)	(6)	(7)	(8)	(9)	(10)	(11)
(1) B1: Small Incentive	0.013	0.135	-0.010	0.030	0.059	-0.020	0.031	0.155	-0.086	0.026	0.177
	(0.206)	(0.189)	(0.077)	(0.067)	(0.045)	(0.199)	(0.070)	(0.474)	(0.057)	(0.082)	(0.087)
	[0.949]	[0.477]	[0.893]	[0.651]	[0.198]	[0.920]	[0.653]	[0.744]	[0.132]	[0.757]	[0.043]
	{1.000}	{1.000}	{1.000}	{1.000}	{0.995}	{1.000}	{1.000}	{1.000}	{0.975}	{1.000}	{0.833}
(2) B2: Large Incentive	0.021	0.520	0.122	0.107	-0.045	0.294	0.029	0.039	0.005	0.048	0.194
	(0.198)	(0.231)	(0.066)	(0.056)	(0.042)	(0.236)	(0.066)	(0.355)	(0.065)	(0.065)	(0.065)
	[0.915]	[0.026]	[0.069]	[0.055]	[0.296]	[0.215]	[0.663]	[0.913]	[0.937]	[0.459]	[0.003]
	{1.000}	{0.786}	{0.919}	{0.895}	{0.998}	{0.995}	{1.000}	{1.000}	{1.000}	{1.000}	{0.347}
(3) B3: Large Grant	0.685	0.697	0.072	0.138	0.016	0.186	0.021	0.001	0.020	0.087	0.213
	(0.205)	(0.246)	(0.095)	(0.069)	(0.047)	(0.235)	(0.058)	(0.352)	(0.070)	(0.061)	(0.090)
	[0.001]	[0.005]	[0.452]	[0.049]	[0.737]	[0.430]	[0.719]	[0.999]	[0.772]	[0.159]	[0.019]
	{0.290}	{0.523}	{1.000}	{0.881}	{1.000}	{1.000}	{1.000}	{1.000}	{1.000}	{0.981}	{0.679}
(4) B4: (Small Incentive)X(Large Grant)	-0.970	-0.727	-0.062	-0.084	-0.051	-0.055	-0.013	0.271	0.256	0.098	0.004
	(0.298)	(0.320)	(0.123)	(0.103)	(0.068)	(0.313)	(0.098)	(0.608)	(0.093)	(0.104)	(0.130)
	[0.001]	[0.025]	[0.614]	[0.418]	[0.452]	[0.860]	[0.897]	[0.656]	[0.006]	[0.351]	[0.976]
	{0.322}	{0.786}	{1.000}	{1.000}	{1.000}	{1.000}	{1.000}	{1.000}	{0.455}	{1.000}	{1.000}
(5) B5: (Large Incentive)X(Large Grant)	-0.693	-0.904	-0.152	-0.267	0.038	-0.422	0.018	0.742	0.001	-0.062	-0.233
	(0.285)	(0.372)	(0.125)	(0.099)	(0.065)	(0.328)	(0.080)	(0.505)	(0.095)	(0.088)	(0.114)
	[0.016]	[0.017]	[0.227]	[0.008]	[0.562]	[0.201]	[0.821]	[0.143]	[0.988]	[0.480]	[0.043]
	{0.729}	{0.729}	{0.995}	{0.593}	{1.000}	{0.995}	{1.000}	{0.976}	{1.000}	{1.000}	{0.833}
(6) Observations	1366	1354	1464	1209	1481	1525	1916	1909	1925	1914	1913
(7) Mean in No Incentive, Small Grant Group	1.401	0.886	0.427	0.277	0.769	1.776	0.792	1.956	0.257	0.587	0.199

NOTES. Table uses sample of children testing anemic at baseline. Children are considered anemic if they have an altitude-adjusted hemoglobin concentration below 120 g/L (per WHO guidelines). Rows 1-5 show estimated coefficients for treatment group indicators and interactions obtained by estimating equation (12) (controlling for the baseline value of the dependent variable, student age, student grade, student sex, number of students in the school, whether the school has a canteen, student teacher ratio, distance to the furthest village served, percent of boarding students, whether the school has implemented the "Free Lunch" policy, county dummy variables, and dummy variables for randomization strata). Standard errors are shown in parentheses, unadjusted p-values are shown in square brackets and p-values adjusted for multiple inference across all tests corresponding to each index are shown in curly brackets. Adjusted p-values were constructed using the free step-down resampling method of Westfall and Young (1993) with 10,000 iterations. *, **, and *** indicate significance at 10%, 5% and 1% based on adjusted p-values.

Table A.8: Nutritional Information to Students and Households, Index Components (Full Sample)

Index:	Information to Households						Information to Students				
Dependent Variable:	Number of school-wide parent meetings attended this semester (Household response)	Number of individual meetings with teacher or administrator this semester (Household response)	School contacted household about student nutrition this semester (Household response)	Household told to give student foods rich in iron (Household response)	Parent reports knowing of anemia (Household response)	Parent correctly identifies foods that can prevent anemia (iron-rich foods) (Household response)	School provided nutritional information to student (Student reported)	Times school spoke with students about nutrition in past semester (Student reported)	Students told to eat meat (Student reported)	Student reports knowing of anemia (Student reported)	School provided information to students on anemia (Student reported)
	(1)	(2)	(3)	(4)	(5)	(6)	(7)	(8)	(9)	(10)	(11)
(1) B1: Small Incentive	0.140	-0.090	-0.034	0.030	-0.012	-0.012	0.084	0.584	0.031	0.098	0.136
	(0.174)	(0.169)	(0.067)	(0.058)	(0.040)	(0.153)	(0.065)	(0.430)	(0.053)	(0.065)	(0.076)
	[0.422]	[0.593]	[0.618]	[0.600]	[0.756]	[0.935]	[0.196]	[0.176]	[0.559]	[0.133]	[0.075]
	{1.000}	{1.000}	{1.000}	{1.000}	{1.000}	{1.000}	{0.966}	{0.959}	{0.996}	{0.932}	{0.854}
(2) B2: Large Incentive	-0.027	0.356	0.097	0.130	0.010	0.296	0.114	0.344	0.103	0.033	0.157
	(0.176)	(0.180)	(0.064)	(0.056)	(0.035)	(0.196)	(0.056)	(0.302)	(0.053)	(0.053)	(0.059)
	[0.878]	[0.050]	[0.132]	[0.023]	[0.772]	[0.133]	[0.041]	[0.256]	[0.054]	[0.531]	[0.008]
	{1.000}	{0.868}	{0.973}	{0.720}	{1.000}	{0.973}	{0.754}	{0.975}	{0.798}	{0.996}	{0.399}
(3) B3: Large Grant	0.612	0.378	0.055	0.089	0.024	0.152	0.094	0.473	0.099	0.084	0.163
	(0.177)	(0.207)	(0.075)	(0.060)	(0.038)	(0.184)	(0.052)	(0.371)	(0.059)	(0.054)	(0.077)
	[0.001]	[0.069]	[0.465]	[0.140]	[0.518]	[0.410]	[0.072]	[0.203]	[0.092]	[0.122]	[0.035]
	{0.174}	{0.915}	{1.000}	{0.973}	{1.000}	{1.000}	{0.854}	{0.966}	{0.887}	{0.929}	{0.722}
(4) B4: (Small Incentive)X(Large Grant)	-0.904	-0.167	-0.024	-0.111	-0.002	-0.232	-0.076	-0.330	0.064	-0.031	0.022
	(0.289)	(0.272)	(0.098)	(0.083)	(0.059)	(0.260)	(0.087)	(0.551)	(0.088)	(0.090)	(0.116)
	[0.002]	[0.539]	[0.803]	[0.186]	[0.970]	[0.374]	[0.383]	[0.550]	[0.470]	[0.730]	[0.853]
	{0.295}	{1.000}	{1.000}	{0.984}	{1.000}	{0.999}	{0.990}	{0.996}	{0.994}	{0.996}	{0.996}
(5) B5: (Large Incentive)X(Large Grant)	-0.424	-0.554	-0.069	-0.125	0.033	-0.332	-0.077	-0.037	-0.094	-0.018	-0.196
	(0.276)	(0.316)	(0.103)	(0.085)	(0.053)	(0.279)	(0.068)	(0.531)	(0.078)	(0.071)	(0.097)
	[0.127]	[0.082]	[0.506]	[0.144]	[0.528]	[0.236]	[0.255]	[0.944]	[0.228]	[0.798]	[0.044]
	{0.973}	{0.934}	{1.000}	{0.973}	{1.000}	{0.993}	{0.975}	{0.996}	{0.969}	{0.996}	{0.758}
(6) Observations	5750	5734	6187	5129	6211	6372	7870	7861	7923	7878	7855
(7) Mean in No Incentive, Small Grant Group	1.434	0.966	0.422	0.255	0.746	1.817	0.692	1.479	0.199	0.539	0.200

NOTES. Table uses full sample of children tested for hemoglobin concentration. Rows 1-5 show estimated coefficients for treatment group indicators and interactions obtained by estimating equation (12) (controlling for the baseline value of the dependent variable, student age, student grade, student sex, number of students in the school, whether the school has a canteen, student teacher ratio, distance to the furthest village served, percent of boarding students, whether the school has implemented the "Free Lunch" policy, county dummy variables, and dummy variables for randomization strata). Standard errors are shown in parentheses, unadjusted p-values are shown in square brackets and p-values adjusted for multiple inference across all tests corresponding to each index are shown in curly brackets. Adjusted p-values were constructed using the free step-down resampling method of Westfall and Young (1993) with 10,000 iterations. *, **, and *** indicate significance at 10%, 5% and 1% based on adjusted p-values.

Table A.9: Child and Household Reported Receipt of Supplements and Iron-Rich Food

Dependent Variable:	Index: Supplements and Food	Sub-index: Supplements	Sub-index: Food	Sub-index: Food at School	Sub-index: Food at Home	Index: Information	Sub-index: Information to Students	Sub-index: Information to Households
	(1)	(2)	(3)	(4)	(5)	(6)	(7)	(8)
Panel A: Impacts Relative to Comparison (No Incentive, Small Grant) Group								
(1) B1: Small Incentive	0.059	0.138	-0.033	-0.08	0.072	0.027	0.062	0.045
	(0.047)	(0.084)	(0.044)	(0.055)	(0.061)	(0.075)	(0.126)	(0.084)
	[0.21]	[0.104]	[0.452]	[0.151]	[0.243]	[0.724]	[0.623]	[0.594]
	{0.572}	{0.534}	{0.599}	{0.676}	{0.700}	{0.984}	{0.982}	{0.809}
(2) B2: Large Incentive	0.114	0.158	0.073	0.039	0.187	0.07	0.111	0.13
	(0.045)	(0.08)	(0.038)	(0.048)	(0.064)	(0.072)	(0.101)	(0.084)
	[0.011]	[0.052]	[0.057]	[0.427]	[0.004]	[0.332]	[0.272]	[0.124]
	{0.199}	{0.456}	{0.498}	{0.676}	{0.149}	{0.926}	{0.906}	{0.699}
(3) B3: Large Grant	0.190**	0.241*	0.138	0.133	0.189	0.162	0.136	0.233
	(0.041)	(0.072)	(0.047)	(0.06)	(0.075)	(0.076)	(0.107)	(0.113)
	[<0.001]	[0.001]	[0.004]	[0.029]	[0.013]	[0.034]	[0.207]	[0.041]
	{0.010}	{0.089}	{0.178}	{0.378}	{0.25}	{0.468}	{0.868}	{0.532}
(4) B4: (Small Incentive)X(Large Grant)	-0.200	-0.269	-0.114	-0.105	-0.15	-0.037	0.19	-0.314
	(0.068)	(0.116)	(0.072)	(0.09)	(0.105)	(0.123)	(0.178)	(0.148)
	[0.004]	[0.021]	[0.116]	[0.243]	[0.157]	[0.766]	[0.288]	[0.036]
	{0.127}	{0.338}	{0.599}	{0.676}	{0.700}	{0.984}	{0.906}	{0.532}
(5) B5: (Large Incentive)X(Large Grant)	-0.248**	-0.289	-0.212	-0.209	-0.292	-0.117	-0.039	-0.356
	(0.064)	(0.106)	(0.067)	(0.079)	(0.11)	(0.108)	(0.151)	(0.146)
	[<0.001]	[0.007]	[0.002]	[0.009]	[0.009]	[0.282]	[0.797]	[0.016]
	{0.032}	{0.206}	{0.128}	{0.233}	{0.205}	{0.915}	{0.984}	{0.398}
(6) Observations	1932	1932	1928	1920	1927	1932	1928	1548
(7) Mean in No Incentive, Small Grant Group	0.031	-0.055	0.039	0.033	-0.053	-0.040	-0.017	-0.082
Panel B: Additional Comparisons								
(8) B1–B2: Effect of Large Incentive vs. Effect of Small Incentive given Small Grant	-0.055	-0.02	-0.106	-0.118	-0.115	-0.044	-0.049	-0.085
	(0.04)	(0.072)	(0.04)	(0.049)	(0.063)	(0.073)	(0.124)	(0.085)
	[0.169]	[0.785]	[0.009]	[0.018]	[0.071]	[0.549]	[0.69]	[0.317]
	{0.572}	{0.86}	{0.217}	{0.315}	{0.537}	{0.984}	{0.984}	{0.809}
(9) B2–B3: Effect of Large Incentive given Small Grant vs. Effect of Increasing Grant Amount	-0.076	-0.083	-0.065	-0.094	-0.002	-0.091	-0.024	-0.102
	(0.042)	(0.074)	(0.046)	(0.061)	(0.068)	(0.085)	(0.114)	(0.105)
	[0.07]	[0.268]	[0.161]	[0.124]	[0.976]	[0.281]	[0.832]	[0.332]
	{0.439}	{0.725}	{0.599}	{0.676}	{0.982}	{0.915}	{0.984}	{0.809}
(10) B2 + B5: Effect of Large Incentive given Large Grant	-0.134	-0.132	-0.139	-0.17	-0.105	-0.047	0.073	-0.225
	(0.043)	(0.066)	(0.051)	(0.059)	(0.074)	(0.08)	(0.111)	(0.117)
	[0.002]	[0.048]	[0.007]	[0.004]	[0.163]	[0.561]	[0.513]	[0.056]
	{0.105}	{0.456}	{0.217}	{0.175}	{0.700}	{0.984}	{0.968}	{0.56}
(11) B3 + B5: Effect of Increasing Grant Amount given Large Incentive	-0.058	-0.049	-0.074	-0.076	-0.102	0.045	0.097	-0.123
	(0.049)	(0.082)	(0.046)	(0.057)	(0.074)	(0.08)	(0.112)	(0.1)
	[0.242]	[0.553]	[0.108]	[0.183]	[0.168]	[0.578]	[0.39]	[0.22]
	{0.572}	{0.86}	{0.599}	{0.676}	{0.700}	{0.984}	{0.939}	{0.797}

NOTES. Table uses sample of children testing anemic at baseline. Children are considered anemic if they have an altitude-adjusted hemoglobin concentration below 120 g/L (per WHO guidelines). Rows 1-5 show estimated coefficients for treatment group indicators and interactions obtained by estimating equation (19) (controlling for the baseline value of the dependent variable, student age, student grade, student sex, number of students in the school, whether the school has a canteen, student teacher ratio, distance to the furthest village served, percent of boarding students, whether the school has implemented the "Free Lunch" policy, county dummy variables, and dummy variables for randomization strata). The dependent variable in each regression is a summary index constructed using the GLS weighting procedure in Anderson (2008). Estimates for the individual components of each index are shown in Appendix Tables 5 and 6. Standard errors are shown in parentheses, unadjusted p-values are shown in square brackets and p-values adjusted for multiple inference are shown in curly brackets. Adjusted p-values were constructed using the free step-down resampling method of Westfall and Young (1993) with 10,000 iterations. *, **, and *** indicate significance at 10%, 5% and 1% based on adjusted p-values. Panel B shows unadjusted and adjusted p-values from tests of linear combinations of coefficients in Panel A.

Table A.10: Heterogeneous Effects by Administrator Prosocial and Intrinsic Motivation

Panel A: Prosocial Motivation

	Anemic at Endline (Hb<120 g/L)			Hemoglobin Concentration (g/L)		
	Low Prosocial	High Prosocial	Difference	Low Prosocial	High Prosocial	Difference
	(1)	(2)	(3)	(4)	(5)	(6)
(1) Small Incentive	-0.178	0.132	0.31**	2.991	-3.59	-6.581
	(0.064)	(0.044)	(0.079)	(1.629)	(1.315)	(2.123)
	[0.006]	[0.003]	[<0.001]	[0.068]	[0.007]	[0.002]
	{0.183}	{0.156}	{0.038}	{0.526}	{0.249}	{0.156}
(2) Large Incentive	-0.154	-0.093	0.061	2.500	1.856	-0.644
	(0.068)	(0.05)	(0.093)	(1.764)	(1.313)	(2.371)
	[0.025]	[0.065]	[0.511]	[0.158]	[0.159]	[0.786]
	{0.316}	{0.436}	{0.621}	{0.653}	{0.653}	{0.840}
(3) Large Grant	-0.237**	-0.082	0.156	5.621	3.601	-2.020
	(0.057)	(0.057)	(0.085)	(1.772)	(1.434)	(2.312)
	[<0.001]	[0.151]	[0.067]	[0.002]	[0.013]	[0.384]
	{0.023}	{0.468}	{0.436}	{0.155}	{0.278}	{0.750}

Panel B: Intrinsic Motivation

	Low Intrinsic	High Intrinsic	Difference	Low Intrinsic	High Intrinsic	Difference
	(1)	(2)	(3)	(4)	(5)	(6)
(4) Small Incentive	-0.078	0.072	0.149	1.828	-3.236	-5.063
	(0.061)	(0.054)	(0.083)	(1.62)	(1.466)	(2.197)
	[0.204]	[0.186]	[0.075]	[0.261]	[0.029]	[0.022]
	{0.774}	{0.774}	{0.570}	{0.764}	{0.440}	{0.416}
(5) Large Incentive	-0.176	-0.129	0.047	2.957	2.499	-0.458
	(0.069)	(0.055)	(0.097)	(1.893)	(1.43)	(2.531)
	[0.012]	[0.02]	[0.628]	[0.12]	[0.082]	[0.857]
	{0.313}	{0.356}	{0.914}	{0.624}	{0.584}	{0.934}
(6) Large Grant	-0.149	-0.181	0.031	4.972	4.131	-0.841
	(0.052)	(0.059)	(0.080)	(1.575)	(1.493)	(2.056)
	[0.005]	[0.003]	[0.694]	[0.002]	[0.006]	[0.683]
	{0.230}	{0.190}	{0.914}	{0.170}	{0.265}	{0.934}

NOTES. Table uses sample of children testing anemic at baseline. Children are considered anemic if they have an altitude-adjusted hemoglobin concentration below 120 g/L (per WHO guidelines). Panel A shows heterogeneous effects of small and large incentives (given small grants) by low and high prosociality scores and Panel B shows heterogeneous effects by low and high intrinsic motivation scores. Both scores are derived from psychological scales in Grant (2008) with individual responses corresponding to each scale combined into indices using the GLS weighting procedure in Anderson (2008). Administrator scores are categorized as low if scores are below the median in the sample at baseline and high if they are above the median. Heterogeneous effects are estimated using equation (19) and interacting treatment dummies with indicators for high baseline prosocial or intrinsic motivation (and controlling for baseline hemoglobin concentration, student age, student grade, student sex, number of students in the school, whether the school has a canteen, student teacher ratio, distance to the furthest village served, percent of boarding students, whether the school has implemented the "Free Lunch" policy, county dummy variables, and dummy variables for randomization strata). Standard errors are shown in parentheses, unadjusted p-values are shown in square brackets and p-values adjusted for multiple inference are shown in curly brackets. Adjusted p-values were constructed using the free step-down resampling method of Westfall and Young (1993) with 10,000 iterations. *, **, and *** indicate significance at 10%, 5% and 1% based on adjusted p-values.

Table A.11: Anemia Status Transitions in the Small Grant Only Group

		Endline	
		Anemic	*Not Anemic*
**Baseline**	*Anemic*	36.40%	63.60%
	Not Anemic	12.10%	87.90%

Table A.12: Bonus Payment Amounts

Group	*Mean*	*SD*
Small Incentive, Small Grant	220.45	195.09
Small Incentive, Large Grant	323.22	332.95
Large Incentive, Small Grant	3,303.33	4,324.39
Large Incentive, Large Grant	3,835.98	4,052.33

Note: Amounts in renminbi exchange rate as of Sept. 2012 was 6.3 USD/RMB).

Appendix B: Theoretical Framework

1 The problem

The school principal's problem is to:

$$\begin{aligned} Max_{\{e,A\}} \quad & w + \theta H - v(e) + S(G - A) \\ \text{s.t.} \quad & w = tH + m \\ & H = f(e, A) \\ & A \leq G \end{aligned}$$

Assuming an internal solution, the problem is equivalent to solving:

$$Max_{\{e,A\}} \quad (\theta + t)f(e, A) - v(e) + S(G - A)$$

The first order conditions are:

$$U_e \equiv (\theta + t)f_e(e, A) - v'(e) = 0 \tag{B.1}$$

$$U_A \equiv (\theta + t)f_A(e, A) - S'(G - A) = 0 \tag{B.2}$$

For simplicity of notation, we suppress the arguments of the functions in the following, so that f_{iij} refers to $f_{iij}(e, A)$ for $i, j = \{e, A\}$, v' refers to $v'(e)$, S' refers to $S'(G - A)$ and similar for the second and third order derivatives of v and S.

The second order conditions are:

$$U_{ee} \equiv (\theta + t) f_{ee} - v'' < 0$$

$$U_{AA} \equiv (\theta + t) f_{AA} + S'' < 0$$

$$|H| \equiv U_{ee} U_{AA} - U_{eA}^2 > 0 \quad \text{, where } U_{eA} = (\theta + t) f_{eA}$$

2 First-order comparative statics

Rewriting B.1 and B.2 as:

$$U_e \Big(e(t, G), A(t, G), t, G \Big) = 0 \tag{B.3}$$

$$U_A \Big(e(t, G), A(t, G), t, G \Big) = 0 \tag{B.4}$$

And differentiating both equations with respect to t, we obtain:

$$U_{ee} \frac{\partial e}{\partial t} + U_{eA} \frac{\partial A}{\partial t} + U_{et} = 0 \tag{B.5}$$

$$U_{Ae} \frac{\partial e}{\partial t} + U_{AA} \frac{\partial A}{\partial t} + U_{At} = 0 \tag{B.6}$$

pplying Cramer's Rule to the above system of equations, we obtain:

$$
\begin{aligned}
\frac{\partial e}{\partial t} &= \frac{-U_{et}U_{AA} + U_{At}U_{eA}}{|H|} \\
&= \frac{-f_e\Big[(\theta+t)f_{AA} + S''\Big] + (\theta+t)f_A f_{eA}}{|H|} > 0
\end{aligned}
$$

$$
\begin{aligned}
\frac{\partial A}{\partial t} &= \frac{-U_{At}U_{ee} + U_{et}U_{Ae}}{|H|} \\
&= \frac{-f_A\Big[(\theta+t)f_{ee} - v''\Big] + (\theta+t)f_e f_{eA}}{|H|} > 0
\end{aligned}
$$

Equivalently but differentiating B.3 and B.4 with respect to G, we obtain:

$$
U_{ee}\frac{\partial e}{\partial G} + U_{eA}\frac{\partial A}{\partial G} + U_{eG} = 0 \tag{B.7}
$$

$$
U_{Ae}\frac{\partial e}{\partial G} + U_{AA}\frac{\partial A}{\partial G} + U_{AG} = 0 \tag{B.8}
$$

And correspondingly, using Cramer's Rule:

$$
\begin{aligned}
\frac{\partial e}{\partial G} &= \frac{-U_{eA}S''}{|H|} \\
&= \frac{-(\theta+t)f_{eA}S''}{|H|} > 0
\end{aligned}
$$

$$
\begin{aligned}
\frac{\partial A}{\partial G} &= \frac{-U_{ee}U_{AG}}{|H|} \\
&= \frac{\Big[(\theta+t)f_{ee} - v''\Big]S''}{|H|} > 0
\end{aligned}
$$

Note that the numerator of $\frac{\partial A}{\partial G}$ can be written as:

$$
|H| - (\theta+t)^2\Big[f_{ee}f_{AA} - f_{eA}^2\Big] + (\theta+t)v''f_{AA} < |H|
$$

Implying that $\frac{\partial A}{\partial G} < 1$, which will be useful in the following[1].

3 Second order comparative statics

To understand when resources and incentives are complements or substitutes, we must find under what conditions the sign of $\frac{\partial H}{\partial t \partial G}$ is positive or negative, respectively. In general the second order comparative statics depend on the third derivatives of $f(e, A)$, $S(G-A)$, and $v(e)$. Because interpreting the third-order derivatives is far from trivial, to make progress we assume that the third derivatives of $f(e, A)$ are zero, but allow $S(G-A)$ and $v(e)$ unrestricted.[2] Using the chain rule on $H = f(e, A)$, we obtain the second-order comparative statics for H:

$$\frac{\partial H}{\partial t \partial G} = \Big[f_{ee}\frac{\partial e}{\partial G}\frac{\partial e}{\partial t} + f_{AA}\frac{\partial A}{\partial G}\frac{\partial A}{\partial t}\Big] + \\ + f_{Ae}\Big[\frac{\partial e}{\partial G}\frac{\partial A}{\partial t} + \frac{\partial e}{\partial t}\frac{\partial A}{\partial G}\Big] + \\ + f_A\frac{\partial A}{\partial t \partial G} + f_e\frac{\partial e}{\partial t \partial G}, \tag{B.9}$$

which could be positive (complements) or negative (substitutes): the first term in brackets is negative, the second term is positive, and its size crucially depends on f_{Ae} and the third and fourth could be positive or negative. In what follows, we study the sign of $\frac{\partial A}{\partial t \partial G}$ and $\frac{\partial e}{\partial t \partial G}$, to help us understand the sign of $\frac{\partial H}{\partial t \partial G}$, and

[1]Note also that $\frac{\partial e}{\partial G} = \frac{-f_{eA}}{f_{ee}}\Big[\frac{\partial A}{\partial G} + v''S''\Big]$, which will also be useful for simplifying the following expressions further.

[2]Note that the third derivatives of $f(e, A)$ include f_{eee}, f_{eeA}, f_{eAA}, f_{AAA} so they are more complex to interpret than the third derivatives of $S(G-A)$ and $v(e)$.

hence ascertain when resources and incentives are complements or substitutes.

To obtain $\frac{\partial A}{\partial t \partial G}$ and $\frac{\partial e}{\partial t \partial G}$, we rewrite B.5 and B.6 as:

$$\Big[(\theta+t)f_{ee}-v''\Big]\frac{\partial e}{\partial t}+(\theta+t)f_{eA}\frac{\partial A}{\partial t}+f_e=0 \tag{B.5'}$$

$$(\theta+t)f_{eA}\frac{\partial e}{\partial t}+\Big[(\theta+t)f_{AA}+S''\Big]\frac{\partial A}{\partial t}+f_A=0 \tag{B.6'}$$

Differentiating with respect to G we get[3]:

$$\begin{aligned}|H|\frac{\partial A}{\partial t \partial G} &= S'''\frac{\partial A}{\partial t}\Big[\frac{\partial A}{\partial G}-1\Big]\Big[(\theta+t)f_{ee}-v''\Big]+(\theta+t)\frac{\partial A}{\partial G}\Big[-f_{ee}f_{AA}+f_{eA}^2\Big]\\ &-v''\left[\frac{(\theta+t)S''\Big[-f_{ee}f_{AA}+f_{eA}^2\Big]+f_{AA}v''S''}{|H|}\right]-v'''\frac{\partial e}{\partial G}\frac{\partial e}{\partial t}(\theta+t)f_{eA},\end{aligned} \tag{B.10}$$

$$\begin{aligned}|H|\frac{\partial e}{\partial t \partial G} &= -(\theta+t)f_{eA}\Big[\frac{\partial A}{\partial G}-1\Big]S'''\frac{\partial A}{\partial t}\\ &+v'''\frac{\partial e}{\partial G}\frac{\partial e}{\partial t}\Big[(\theta+t)f_{AA}+S''\Big]+(\theta+t)\frac{\partial e}{\partial G}\Big[-f_{AA}f_{ee}+f_{eA}^2\Big]+\frac{f_{eA}v''(S'')^2}{|H|}.\end{aligned} \tag{B.11}$$

The expressions $(B.10)$ and $(B.11)$ are complex but they can be understood if we take into account that:

1. $|H|>0$,

[3]And assuming that the third derivatives of f are null as previously discussed

2. the terms that include S''' have the same sign as the sign of S''',

3. the terms that include v''' have the opposite sign as the sign of v''',

4. all other terms are negative, except $\frac{f_{eA}v''(S'')^2}{|H|}$ in $(B.11)$, which can be null or positive.

Given the above, the conditions that are more favorable to complements $\frac{\partial H}{\partial t \partial G} > 0$ are $S''' > 0$, $v''' < 0$, and a large value of f_{eA}. Analogously, the conditions that are more favorable to substitutes $\frac{\partial H}{\partial t \partial G} < 0$ are: $S''' <= 0$, $v''' >= 0$, and a small value of f_{eA}.

Appendix C: Anemia Information Video Script Translation

Hello! My name is Dr. Wang and today I'm going to talk to you about something called anemia. Did you know that around 40% of children in rural areas of China are anemic? This is a problem for principals like yourself because anemic children do worse in school than healthy children!

So, what is anemia? Anemia is a disease that makes it difficult for the oxygen you breathe in to get to your muscles and brain. Without oxygen in your brain, you are unable to think clearly. So how do we fix this? Well, by eating lots of iron-rich foods! Foods like meat, beans and tofu will help turn a sickly, anemic person into a stronger, smarter healthy person!

How can you tell if any students in your school are anemic? One of the most troublesome things about anemia is that there are often no symptoms. A healthy looking child may still be anemic! At times, there may be signs that children are anemic. Anemic children sometimes get headaches and complain about being dizzy. Anemic children sometimes feel chronically tired. They might be pale. Anemic students may have a hard time paying attention in class. Many anemic children end up skinner and shorter than they should be. BUT SOME DO NOT. Again: there may be children that do not have any of these symptoms, but who still have anemia. And, when students have anemia, this puts them at a disadvantage compared to their healthier classmates.

In fact, researchers have studied anemia and its effect on grades in Shaanxi, Ningxia, Gansu and Qinghai. In ALL of the studies, it was found that children with anemia perform worse than their classmates in school. They get worse grades, and perform worse on standardized intelligence tests. When anemic students are treated for

anemia, their grades in school have been shown to increase by up to five points! This is the difference between a C+ (75 points out of a 100) and a B (80 points or more)!

There is some Good News, however. All of these problems can be easily fixed, just by treating anemia in your school.

Researchers have found that around one-third of children in your area have anemia. If you suspect that some students in your school might be anemic, don't worry—anemia is easy to treat! To fight and prevent anemia—and improve student performance in class—you can just make a few simple changes in your students' diet to make sure they are getting enough iron and eating a balanced diet.

What foods have iron? MEAT. Chicken, beef, lamb and pork—all animal meats are great sources of iron. Eggs and milk are healthy foods, but they don't have any iron!

Some other foods have iron too, but it is harder for children to absorb iron from non-meat sources. Meat is the best source of iron. Other sources of iron are pumpkin or squash seeds, potato skins, tofu and soy products, peanuts, and beans. If you want to add these foods to your students' diet, make sure you also add lots of fruits and vegetables so that the children can absorb the iron into their bodies. Vegetables like green peppers, chili peppers, and red dates are all good sources of vitamin C, which children need to absorb iron. Did you know that adding a red date or other source of vitamin C to your students' meal can boost their rate of iron absorption by as much as 300%?

Eating a balanced diet each day is important for overcoming anemia, so feed your child lots of meat and vegetables in *addition* to staples like plain rice, noodles, and buns. Eating staples may make children *feel* full, but they don't contain any iron and have few

other nutrients, so by themselves they are not healthy. Staples should be paired with meat or vegetables to make a healthy meal.

Remember, adding ONLY more fruits and vegetables to your students' meals is good, but not enough.

Adding ONLY more grains to your students' meals is good, but not enough.

A balance of meat, fruits and vegetables is key to keeping students in your school healthy and ready to learn!

Exactly how much meat does a child need to eat to stay healthy? He should eat half a jin of meat each day. How much is that? Make a fist with your hand. Every day your students should eat about two fists of meat. More specifically, each student could eat:

- ½ jin of pork (two and a half fists)
- 1/3 jin of chicken (one and a half fists)
- ¾ jin of lamb (four fists)
- 4/5 jin of tofu (two fists)
- 4/3 jin of beans (one bowl)

Keep in mind that this is how much iron a child should consume in a full day, not just in a single meal. Since many children eat meals at both home and school, you may have to share the responsibility for child nutrition with students' parents. You can talk to them about student nutrition and the importance of a balanced meal.

Even if you can't provide students with as much meat as they need, remember that some meat is better than none at all!

Now let's make some healthy meal combinations! Here are some examples of a healthy school lunch:

- Chicken and green pepper
- Pork and chopped cabbage
- Tofu and chili peppers
- String beans with ground pork

In addition to supplementing school meals with meat, vegetables and fruit, there are other things you can do to reduce anemia in your schools. Some of these options might be cheaper or easier than buying meat everyday, and can be just as effective!

One option is to give students a daily multivitamin with iron. A single vitamin tablet has as much iron as a whole dish of pork! Vitamins are easy to dispense – other principals in your area have tried it and found that students in their schools became more energetic and performed better in school. A daily multivitamin helps prevent anemia and especially helps children who are already anemic and most at-risk for learning problems. Follow the recommended dosages for children on the bottle, and be sure to provide clean drinking water for students to use when taking vitamins. Many manufacturers now make chewable vitamins for children. These are easy to take, don't require any water, and kids often enjoy the taste!

Vitamins may not be readily available at your local grocery store, but we can help you purchase them easily.

Another way to reduce anemia in your school is to cook school meals with foods that are fortified with iron. Some special types of soy sauce and flour have extra iron added. These are cheap, and taste the same as regular soy sauce and flour. Fortified foods are

often difficult to find at your local grocery store, but we can help you purchase them easily.

You may also choose to tell parents about anemia and why it is important. Tell them that their children might not be doing as well in school as they should be, and share the strategies you have learned to reduce anemia. Explain the importance of a balanced diet. Persistent efforts from teachers and principals to talk to parents is very important, and there are many successful examples of this. Most parents are very concerned about the well-being of their children. However, it is not an easy task to convey key nutrition and health information to parents! Giving parents pamphlets or letters about anemia and how to prevent anemia is not always effective. Some parents may not receive the information, some may not understand it, and some may think they do not have the economic means to change their dietary habits.

Instead, try holding meetings at school with parents to communicate information directly. Frequently follow up to check in about progress and to answer any questions parents might have.

Remember that the best strategy for reducing anemia will depend on local conditions at your school—and no one knows your school better than you do! Can you think of other ways to reduce anemia in your school? Be creative! You know your school and students best, so you may be able to think of even better strategies for keeping your students healthy.

Why is it so important to address the anemia problem in your school? Well, researchers in Shaanxi, Ningxia, Gansu and Qinghai found that anemic children who took a daily multivitamin with iron saw a big improvement in their test scores. This was

because as their anemia was treated, they grew stronger, could think more clearly, and got better grades. Eating meat does the same thing!

Whichever option you choose, remember that it is your responsibility to make sure your students are eating healthily everyday. They will grow taller, think more clearly, and get better grades so that they can go on to high school and college. Help give China's children a better future!
